# Domain-generalizable oral cancer classification: bridging multi-center histopathological datasets

**DOI:** 10.3389/fmed.2026.1951350

**Published:** 2026-09-09

**Authors:** Ziying Hua, Yushi Zhou

**Affiliations:** 1 School of Stomatology, Harbin Medical University, Harbin, Heilongjiang, China; 2 First Affiliated Hospital of Kunming Medical University, Kunming, Yunnan, China

**Keywords:** computational pathology, deep learning, domain generalization, oral squamous cell carcinoma, stain invariance

## Abstract

Deep models for oral squamous cell carcinoma (OSCC) detection from haematoxylin-eosin histopathology degrade sharply across laboratories, scanners and magnifications, yet the target centre is usually unavailable during training, making generalization to unseen domains the decisive requirement for reliable screening. We cast multi-centre OSCC classification as a domain-generalization problem and propose SICA (Style-Invariant Consistency Alignment), a single objective combining three established invariance mechanisms: style randomisation of channel-wise feature statistics, a stain-consistency term forcing two independently stained views of each image to agree in embedding and prediction space, and a class-conditional feature alignment that preserves the decision boundary. Two of them rely only on paired stain views, so SICA remains effective with a single source domain, where classical cross-domain alignment collapses to plain risk minimisation. On a reproducible leave-one-domain-out benchmark spanning a genuine cross-magnification shift and a controlled multi-centre stain-shift simulation, SICA is compared against nine baselines over eight metrics. Among the ten algorithms sharing a common ImageNet backbone it attains the best and most stable out-of-domain AUROC (90.6% on the multi-centre protocol), the best worst-case centre, and a balanced operating point better calibrated than empirical risk minimisation. With confidence intervals, paired tests and multiplicity correction, that margin over the strongest baselines proves consistent but not statistically decisive on five simulated centres. Under the same protocol, a frozen pathology foundation model closes part of the stain gap but not the scale gap, and the proposed objective improves it further (92.3% to 93.4% AUROC on identical frozen features), so better representations and explicit invariance are complementary. An audit of the public corpus uncovered 28 byte-identical images filed under contradictory labels, removed before re-running the benchmark under a group-aware, leakage-controlled protocol. Finally, on a genuinely independent 150-patient cohort we report a negative result: every method, ours included, falls to chance-level discrimination, because the binding constraint is field-of-view scale rather than stain. Stain invariance is therefore necessary but not sufficient, and a simulated stain-shift benchmark must not be read as an estimate of deployment performance.

## 1 Introduction

Oral squamous cell carcinoma (OSCC) is among the most common malignancies of the head and neck and a major cause of cancer mortality worldwide, with prognosis depending strongly on how early the disease is detected (1–3). Histopathological examination of haematoxylin–eosin (H&E) stained tissue is the diagnostic reference standard, but manual assessment is time-consuming, requires scarce specialist expertise, and is subject to appreciable inter- and intra-observer variability (4–6). Deep learning has therefore attracted intense interest as a means of automating the discrimination of malignant epithelium from normal oral mucosa (7–9), promising to reduce pathologist workload, standardise reporting and extend screening capacity. Under a single-cohort setting—training and test images sharing one staining and scanning protocol—such models already report high accuracy and recall (10–12), confirming that the morphological signatures of OSCC are learnable directly from H&E appearance.

The obstacle to clinical adoption is that real deployment is inherently multi-centre (13, 14). Tissue processed and digitised at different laboratories varies in staining reagents, section preparation, scanner hardware and magnification, inducing a pronounced covariate shift between institutions (15, 16); the dominant nuisance factor is the H&E colour appearance itself, compounded by differences in field of view and cellular scale. A model trained at one centre consequently degrades sharply at another, even though the diagnostic task is unchanged (17). Critically, the target centre’s data are usually *not* available during training—a new hospital, scanner or protocol cannot be observed in advance—so the classifier must generalise to unseen domains without adaptation. Robustness to such unseen stain (18), scanner and magnification shifts, rather than in-distribution accuracy, is thus the decisive requirement for trustworthy OSCC screening across sites, and it is the problem we address.

Existing remedies each address only part of this challenge. Stain normalisation and stain augmentation operate almost entirely at the input and colour level (19–22): they canonicalise or perturb H&E appearance without compelling the internal representation to become invariant, and normalisation can fail when the deployment appearance departs from its reference (23, 24). General domain-generalization strategies based on cross-domain feature alignment, adversarial training, invariant prediction or worst-case optimisation require *multiple* labelled source domains to be active (25, 26), and therefore collapse to ordinary risk minimisation in the common single-source regime (27), while aligning the marginal feature distribution can additionally blur the boundary between classes. Consistency and multi-view learning offer a label-free route to invariance (28), but have largely been used for representation pre-training rather than as a supervised regulariser targeted at cross-centre OSCC generalization (29–31), and are seldom combined with class-conditional alignment. Finally, prior evaluations are fragmented: they typically report either a narrow real shift or a purely synthetic one, rarely benchmark a wide range of domain-generalization algorithms under a unified protocol for OSCC (32, 33), and often overlook the severe OSCC/Normal class imbalance and the probability calibration that matter for screening (34). Therefore, our research addresses these gaps as follows:

1. We propose *SICA* (Style-Invariant Consistency Alignment), a single training objective that combines three complementary and individually well-established invariance mechanisms—style randomisation of channel-wise feature statistics inside the backbone, a stain-consistency term forcing two independently stained views of every image to agree in embedding and prediction space, and a class-conditional feature alignment matching second-order statistics within each class across source domains. Because two of these depend only on the paired stain views of a single image, SICA remains effective in the single-source regime where classical cross-domain alignment degenerates.
2. We formulate multi-centre OSCC screening as a domain-generalization problem and build a reproducible, multi-protocol leave-one-domain-out benchmark pairing a genuine cross-magnification shift with a controlled multi-centre stain-shift *simulation*, comparing ten algorithms under identical training, class-imbalance handling and model selection across eight metrics including calibration. We are explicit that the virtual centres are synthesised from a single public cohort and are not a substitute for real inter-institutional validation: they isolate the colour axis under otherwise identical content, which makes the comparison between algorithms clean, but they do not reproduce the joint variation in optics, section thickness, fixation and protocol that separates real laboratories. We therefore also evaluate every trained model, without adaptation, on a genuinely independent 150-patient cohort acquired at other institutions and at a different magnification (Section 4.12), and report the outcome—a failure of all methods, with a diagnosis—rather than only the controlled comparison.
3. We audit the public dataset itself and report that 28 of its 1,224 images are byte-identical duplicates filed under *both* class labels, together with a set of near-duplicates; we release the audit, repeat the benchmark on a de-duplicated corpus with group-aware domain assignment, and quantify how much the reported performance depends on this correction.
4. We benchmark a frozen self-supervised pathology foundation encoder against a matched-architecture ImageNet control under the same protocol. Pathology pre-training helps against stain shift but not against scale shift, most of its benefit is realised only when the head is trained to be stain-robust, and the proposed objective improves the foundation model further on identical frozen features—so representation quality and explicit invariance are complementary rather than alternatives.
5. We show that SICA delivers the best and most stable out-of-domain performance among the ten methods sharing a common backbone—raising worst-case robustness on the hardest, faint-staining centre above the average of eight of the nine baselines, producing a well-balanced sensitivity–specificity operating point with calibration improved over empirical risk minimisation, and transferring without target-domain data or tuning. We report this with confidence intervals, paired significance tests and multiplicity correction, and state plainly which margins are supported: the mean gain over the strongest baselines is not, on its own, statistically decisive, and the case for SICA rests on the consistency of the improvement—best mean rank, smallest variance, best worst-case centre—rather than on a single average.

The remainder of this paper reviews related work, details the SICA framework and its training objective, describes the two evaluation protocols, and presents a comprehensive experimental comparison and analysis.

## 2 Related work

### 2.1 Deep learning for oral cancer histopathology

Automated analysis of H&E whole-slide and patch images has become a central tool for OSCC research (27), and convolutional networks have been widely adopted to distinguish malignant epithelium from normal oral mucosa (8–12), to grade dysplasia and to localise tumour regions (7, 35, 36). Most of this literature follows a single-cohort paradigm: a model is trained and evaluated on images acquired under one staining and scanning protocol, and reports strong accuracy under that fixed distribution. Systematic reviews of the field note that this design dominates and that evaluation quality is frequently its weak point (37–39). Such studies establish that the morphological cues of OSCC—nuclear pleomorphism, altered chromatin, disrupted architecture—are learnable from H&E appearance, but they assume that training and test images come from the same laboratory. When that assumption breaks, the reported performance rarely transfers, which motivates treating the problem as one of generalization rather than of in-distribution classification.

### 2.2 Stain and scanner variability in computational pathology

Variation in staining reagents, section thickness, scanners and magnifications induces a substantial covariate shift that degrades deep models across centres (15, 16, 40), and two remedies dominate. Stain normalisation canonicalises the colour appearance of every image toward a reference, for example by colour deconvolution (41) and re-projection, as popularised by Macenko-style estimation of stain vectors from the optical-density plane (19, 20, 23, 42); the other two classical normalisers we compare against are the LAB-space colour transfer of Reinhard et al. (43) and the structure-preserving sparse factorisation of Vahadane et al. (44). Stain augmentation instead perturbs H&E colour during training, most commonly by random affine jitter of the stain channels in a deconvolved colour space (21, 22, 24, 45, 46), so that the network becomes insensitive to appearance rather than relying on a fixed palette. Both families operate almost entirely at the input level and address only the colour axis; normalisation can moreover fail when the deployment appearance departs markedly from the reference (47), and augmentation does not explicitly encourage the internal representation to be invariant.

### 2.3 Domain generalization

Beyond stain-specific fixes, the machine-learning community has proposed general strategies that assume several labelled source domains and aim to learn a predictor that transfers to an unseen target (25, 26). Feature-alignment methods reduce the discrepancy between source domains in representation space, by matching second-order statistics (48) or minimising a kernel-based maximum mean discrepancy (17, 49); adversarial approaches train features to be indistinguishable across domains through a gradient-reversal discriminator (50); causally motivated objectives seek a representation on which the optimal classifier is invariant across environments (51); and distributionally robust optimisation minimises the worst-case source risk (52). A parallel augmentation-based line perturbs the training distribution directly, either by convex interpolation of cross-domain samples (53) or by mixing channel-wise feature statistics to synthesise novel styles on the fly (18, 54–56). The latter operator is inherited from adaptive instance normalisation (AdaIN) (57), itself built on instance normalisation (58): AdaIN replaces the channel-wise statistics of a content feature map with those of a *style* image, whereas MixStyle replaces them with a convex combination of the statistics of two training samples, turning the same operator into a stochastic augmentation. We use the MixStyle form unmodified. A recurring limitation of the alignment, adversarial and robustness families is that their objectives require *multiple* active source domains: with a single source they have no cross-domain signal and reduce to empirical risk minimisation.

### 2.4 Consistency regularisation and multi-view representation learning

Related to our approach is the idea of enforcing agreement between different views of the same input, which underpins much of modern self-supervised and semi-supervised learning (28): two stochastically transformed versions of an image are encouraged to map to consistent embeddings or predictions, often with stop-gradient or asymmetric predictors that prevent collapse. In pathology the natural transformation is a colour perturbation—two independently stained views of the same tissue share content but differ in H&E appearance—so requiring agreement provides a direct, label-free signal for stain invariance. This has mostly been explored for representation pre-training rather than as a supervised regulariser targeted at cross-centre generalization (29–31, 59), and is rarely combined with explicit alignment of class-conditional feature distributions. The specific ingredients we reuse are the $\ell_{2}$-normalised negative-cosine objective of BYOL (60) and its symmetric stop-gradient form in SimSiam (61), and, in prediction space, the temperature-softened targets of knowledge distillation (62) as used by the consistency regularisers of the semi-supervised literature (63–66).

### 2.5 Pathology foundation models

A development that post-dates most of the domain-generalization literature is the arrival of pathology *foundation models*: vision encoders pre-trained self-supervised on tens of thousands of whole-slide images spanning many organs, laboratories, scanners and staining protocols. UNI (67), Virchow and Virchow2 (68, 69), Phikon (70), CHIEF (71) and the vision–language model CONCH (72) all report strong transfer with frozen features and little supervision. Because their pre-training corpora are themselves multi-centre, such encoders can be expected to be intrinsically more robust to stain and scanner variation than an ImageNet-pretrained convolutional network, and this reframes the domain-generalization question: how much of the cross-centre gap is closed simply by a better representation, and how much requires an explicit invariance objective? The evidence is not one-sided—an independent clinical benchmark of public pathology foundation models reports that no single model dominates and that performance still varies appreciably across sites (73)—so the two lines are complementary rather than substitutes. We therefore include frozen pathology-foundation-model baselines, together with a matched-architecture ImageNet control, in Section 4.13, and treat our invariance objective as something that can be applied *on top of* whichever encoder is available.

### 2.6 Positioning of this work

SICA sits at the intersection of these lines. Rather than choosing between input-level colour handling and feature-level invariance, it combines three complementary mechanisms—each individually established, and attributed in Section 3.1—in a single objective: style randomisation of channel-wise feature statistics, a stain-consistency term on two colour views of every image, and a class-conditional alignment that matches second-order statistics *within* each class so as not to collapse the decision boundary. Two of the three depend only on the paired views of a single image and therefore remain effective in the single-source regime where classical alignment degenerates. Finally, whereas most prior evaluations report either a real but narrow cross-magnification shift or a purely synthetic one, we assess every method under two complementary leave-one-domain-out protocols (32, 33, 74) and on an independent external cohort.

## 3 Methodology

### 3.1 Relation to prior work: what is reused and what is new

Before describing the method we state plainly which of its parts are taken from the literature, because each of the three mechanisms of SICA is an established technique rather than a new operator. Style randomisation is MixStyle (18, 54), used unmodified, whose style operator descends from adaptive instance normalisation (57) and instance normalisation (58) and whose Beta-distributed interpolation coefficient comes from mixup (53). The embedding-space consistency term is the $\ell_{2}$-normalised negative-cosine objective of BYOL (60) in the symmetric stop-gradient form of SimSiam (61); the prediction-space term is the temperature-softened, $T^{2}$-rescaled divergence of knowledge distillation (62) applied as a two-view consistency regulariser in the manner of the semi-supervised literature (63–66), with the symmetrisation of deep mutual learning (75, 76) and the linear ramp-up schedule of (64, 65). The alignment term is the Deep CORAL discrepancy (48, 77) augmented with a first-order mean-matching term, which makes it an order-two central moment discrepancy (78, 79) rather than CORAL proper; conditioning such an alignment on the class label so that it does not collapse the decision boundary is likewise an established idea (80–83). The colour operator that generates our two views is the haematoxylin–eosin–DAB (HED) stain augmentation of Tellez et al. (45, 46) in the colour-deconvolution space of Ruifrok and Johnston (41), and our multi-source baselines follow the DomainBed protocol (84).

Our contribution is therefore not a new loss primitive but (i) the particular composition of these three invariances into one objective, (ii) the choice of an *H&E stain* perturbation as the view-generating transform, which turns a generic consistency regulariser into a targeted stain-invariance regulariser, (iii) the observation and empirical demonstration that this composition keeps working in the single-source regime where the alignment, adversarial and worst-case families degenerate to empirical risk minimisation, and (iv) the multi-protocol, leakage-audited OSCC benchmark with calibration and inferential statistics on which all ten algorithms are compared. Wherever a claim of novelty is made in what follows, it is a claim at this level of composition and evaluation.

### 3.2 Overall model architecture

**Figure 1:**
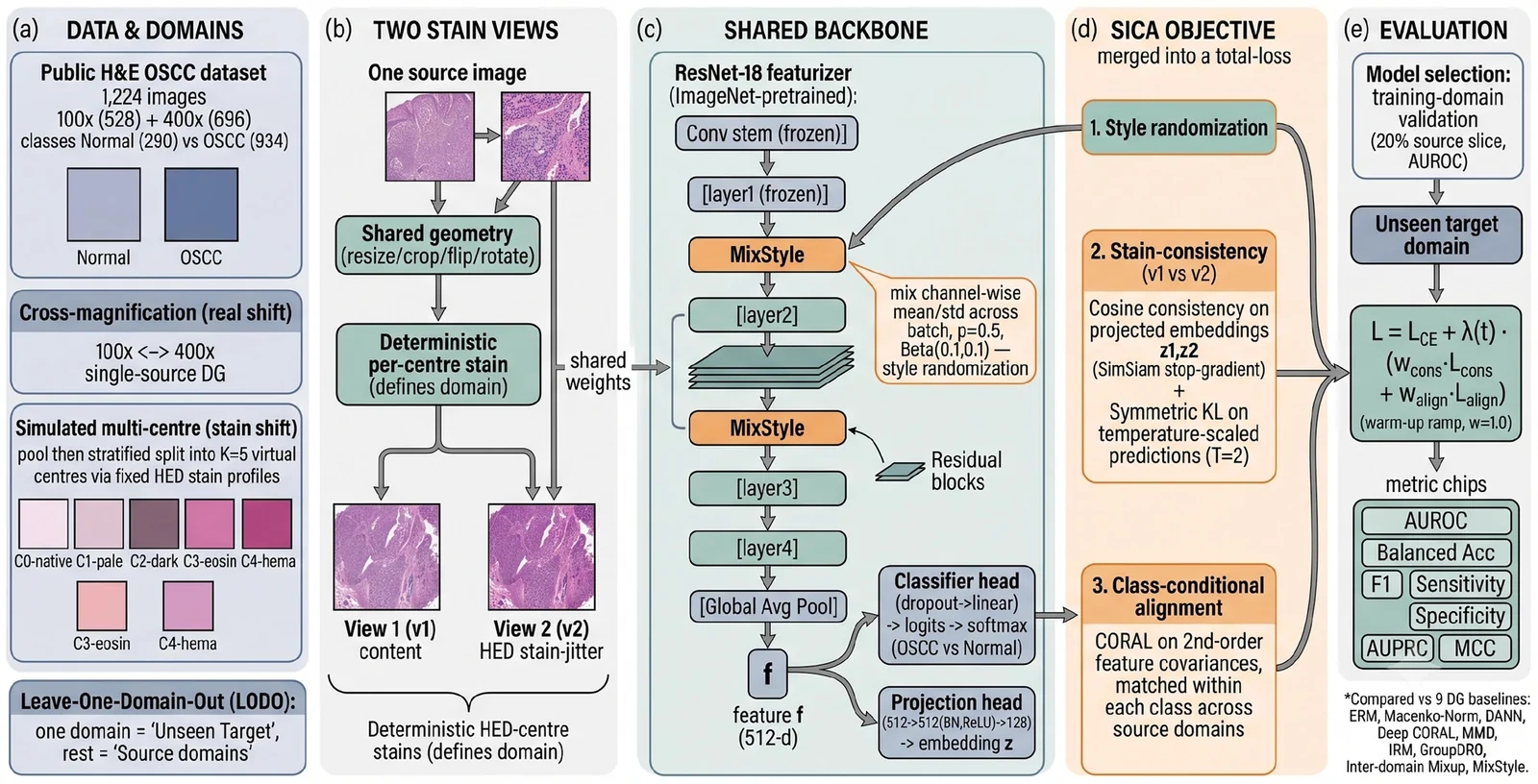
Overview of the SICA framework for domain-generalizable OSCC histopathology classification. **(a)** Data and domain construction under two leave-one-domain-out protocols: a real cross-magnification shift (100× vs. 400×) and a controlled multi-centre stain shift obtained by recolouring pooled images into K=5 virtual centres with fixed HED stain profiles. **(b)** Two-view pipeline: shared geometric augmentation, a deterministic per-centre stain that defines the domain, then two stain-jittered views with identical content but different H&E appearance. **(c)** Shared ImageNet-pretrained ResNet-18 featurizer with MixStyle (54) between the early stages, feeding a classification head and a projection head. **(d)** The training objective, coupling style randomisation, cross-view stain-consistency and class-conditional alignment. Its components are prior work, cited where introduced: MixStyle (54), the SimSiam-style symmetric stop-gradient objective (60, 61), temperature-scaled distillation (62) and Deep CORAL (48); see Section 3.1. **(e)** Evaluation: the checkpoint is selected by training-domain validation and assessed on the unseen target across eight metrics against nine baselines.

The overall architecture of SICA (Style-Invariant Consistency Alignment) is illustrated in Figure 1. The model is trained on a set of source domains and must generalise to an unseen target domain without accessing any of its data. Following a leave-one-domain-out (LODO) protocol, one domain is held out as the unseen target while the remainder serve as sources. To probe the two nuisance factors that dominate computational pathology, domains are defined in two complementary ways (Figure 1a): a real cross-magnification shift, in which 100× and 400× images form separate domains, and a controlled multi-centre stain shift, in which all images are pooled and, stratified by class and native magnification, partitioned into K virtual centres, each recoloured by a fixed haematoxylin–eosin–DAB (HED) colour-deconvolution (41) stain profile, so that centres differ only in staining appearance.

Each image first undergoes stain-agnostic geometric augmentation (resizing, cropping, flipping, rotation) shared across views, then the deterministic per-centre stain that establishes its domain, and finally two independent random HED jitters (Figure 1b). The two resulting views share tissue content and spatial layout but differ in H&E colour, providing the paired signal that drives the stain-invariance objective. Both views are encoded by the same ImageNet-pretrained ResNet-18 in a Siamese manner (Figure 1c); the stem and first residual stage are frozen to preserve generic low-level filters and stabilise training on a moderately sized dataset, MixStyle modules between the early stages perturb channel-wise feature statistics during training and are disabled at inference, and global average pooling yields a 512-dimensional feature feeding a classification head and a projection head used only by the consistency objective.

The three invariance mechanisms are optimised jointly (Figure 1d): style randomisation inside the featurizer; a stain-consistency term that forces the two views to agree both in the projected embedding space and, through a temperature-softened symmetric divergence, in prediction space; and a class-conditional alignment that matches second-order feature statistics within each class across source domains, so that domain-induced offsets are suppressed without collapsing the boundary between normal and malignant tissue. The two regularisers are ramped up through a warm-up schedule so that a reliable feature space is established before invariance is enforced. Because the dataset is markedly imbalanced, training uses class-balanced sampling. Model selection follows the standard training-domain validation protocol—a stratified 20% slice of the sources, never the target, with the best validation AUROC retained—and the selected model is evaluated on the untouched target domain (Figure 1e) across eight metrics against nine established baselines.

### 3.3 Problem formulation and domain-generalization setup

We cast oral squamous cell carcinoma (OSCC) screening as a domain-generalization (DG) problem over haematoxylin–eosin (H&E) histopathology images, where each image is associated with a binary label y∈Y={0,1} denoting normal mucosa (y=0) or OSCC (y=1). Let $D=\{D_{1},\ldots ,D_{M}\}$ be a set of M domains, where every domain $D_{k}$ is a distribution over the joint space X×Y and successive domains differ in their acquisition conditions, such as magnification, scanner, or staining protocol. Under a leave-one-domain-out (LODO) protocol, a single domain $D_{t}$ is withheld as the *unseen target* while the remaining domains $S=D\setminus \{D_{t}\}$ act as *sources*, and the learner may access data only from S during training. Our goal is to learn a predictor $h=C_{\phi }\circ F_{\theta }$, composed of a feature extractor $F_{\theta }$ and a classifier $C_{\phi }$, that minimises the expected target risk without ever observing the target distribution, as stated in Equation 1,

$$\min_{\theta ,\phi }\;E_{(x,y)∼D_{t}}\;\left[\ell \left(h(x),y\right)\right],\;h(x)=C_{\phi }\left(F_{\theta }(x)\right),$$ (1)

where ℓ(⋅,⋅) is a classification loss and the optimisation is carried out purely on S. Because the two dominant nuisance factors in computational pathology are field-of-view scale and H&E colour appearance, we instantiate D under two complementary definitions—a real cross-magnification shift and a controlled multi-centre stain shift—so that the same model can be stress-tested against both a genuine and a colour-isolated distribution shift.

### 3.4 Stain-aware domain construction and two-view generation

To manipulate staining appearance in a physically meaningful way, we operate in the haematoxylin–eosin–DAB (HED) colour-deconvolution space of Ruifrok and Johnston (41), in which an RGB intensity $I\in [0,1{]}^{3}$ is mapped to per-stain concentrations through an invertible transform $\Phi_{\mathrm{HED}}$ and recovered by its inverse $\Phi_{\mathrm{HED}}^{-1}$. A stain edit is expressed as a per-channel affine operation on the H and E concentrations followed by a global brightness scaling,

$$T_{\alpha ,\beta ,\gamma }(I)=\gamma \cdot \Phi_{\mathrm{HED}}^{-1}\;\left(\alpha ⊙\Phi_{\mathrm{HED}}(I)+\beta \right),$$ (2)

where $\alpha ,\beta \in R^{3}$ scale and bias the stain channels, γ>0 is a brightness factor, and ⊙ denotes the element-wise product. For the controlled multi-centre benchmark, all images are pooled and, stratified by class and native magnification, partitioned into K virtual centres; each centre k is assigned a *fixed* profile $\left(\alpha^{\left(k\right)},\beta^{\left(k\right)},\gamma^{\left(k\right)}\right)$ and every image belonging to it is recoloured deterministically by $T_{\alpha^{\left(k\right)},\beta^{\left(k\right)},\gamma^{\left(k\right)}}$, so that centres share identical content and differ only in colour statistics and the domain variable isolates stain shift. During training we additionally draw two independent random stain views of every image with the HED stain augmentation of Tellez et al. (45, 46), by sampling the affine parameters as $\alpha_{c}∼U(1-\sigma ,\;1+\sigma )$ and $\beta_{c}∼U(-b,\;b)$ for the H and E channels together with a brightness factor γ∼U(1−η,1+η), yielding a pair $\left({\tilde{x}}^{\left(1\right)},{\tilde{x}}^{\left(2\right)}\right)$ that shares geometry and content but differs in H&E appearance. Concretely, each sample first undergoes shared, stain-agnostic geometric augmentation (resizing, random cropping, flipping, and rotation), then the deterministic per-centre stain that fixes its domain, and finally two independent HED jitters, so that the resulting views form the paired signal exploited by the stain-invariance objective of Section 3.7.

#### 3.4.1 Exact specification of the virtual centres

The pooled corpus is partitioned once, before any train/test split, by a permutation drawn from a NumPy PCG64 generator seeded with ${\mathrm{seed}}_{\mathrm{centre}}=0$ and applied inside every (class × native-magnification) stratum, followed by round-robin assignment; centres are therefore balanced in class and magnification content to within one image, and the assignment is identical for every method and seed. Each centre then receives a fixed profile $\left(\alpha_{H},\alpha_{E};\beta_{H},\beta_{E};\gamma \right)$: C0-native (1.00,1.00;0,0;1.00), C1-pale (0.80,0.82;−0.03,−0.03;1.10), C2-dark (1.20,1.18;0.05,0.04;0.92), C3-eosin (0.95,1.25;0,0.06;1.00) and C4-hema (1.28,0.90;0.06,−0.02;0.97). These span the pale, dark, eosin-rich and haematoxylin-rich appearances routinely observed between laboratories, act only on the H and E channels (the residual DAB channel keeps $\alpha_{3}=1,\beta_{3}=0$), and—crucially—depend on the centre index alone: the same transform is applied to every image of a centre irrespective of its label, so it cannot inject a class-specific cue by construction. The training-time jitter of Equation 2 uses $\alpha_{c}∼U(1-\sigma ,1+\sigma )$, $\beta_{c}∼U(\;-b,b)$ on the H and E channels and γ∼U(1−η,1+η), at σ=b=η=0.05 with application probability $p_{\mathrm{jit}}=0.8$ per view; the strong-augmentation baseline of Section 4.11 uses σ=b=0.20, η=0.15 and $p_{\mathrm{jit}}=1$. Every experiment is repeated with training seeds {0,1,2}, which jointly seed the initialisation, the class-balanced sampler, the augmentation stream and the source train/validation split.

#### 3.4.2 Order of operations relative to the split

The pipeline is ordered so that no augmentation can leak across the split: (i) the centre partition is computed on the manifest; (ii) one centre is withheld as the unseen target; (iii) a stratified 20% slice of the sources is held out for model selection; (iv) only then, and only inside the training loop, are images transformed—shared geometric augmentation, the deterministic per-centre stain, an optional stain normalisation, and finally the two independent HED jitters. Steps (i)–(iii) operate on identifiers only; the stochastic transforms of step (iv) are never cached, never applied to the target domain and never used to fit any statistic. Stain normalisers are fitted to a fixed reference appearance rather than to the pooled corpus, so no target-domain information enters through preprocessing.

#### 3.4.3 Morphology is preserved by construction, and empirically

**Figure 2:**
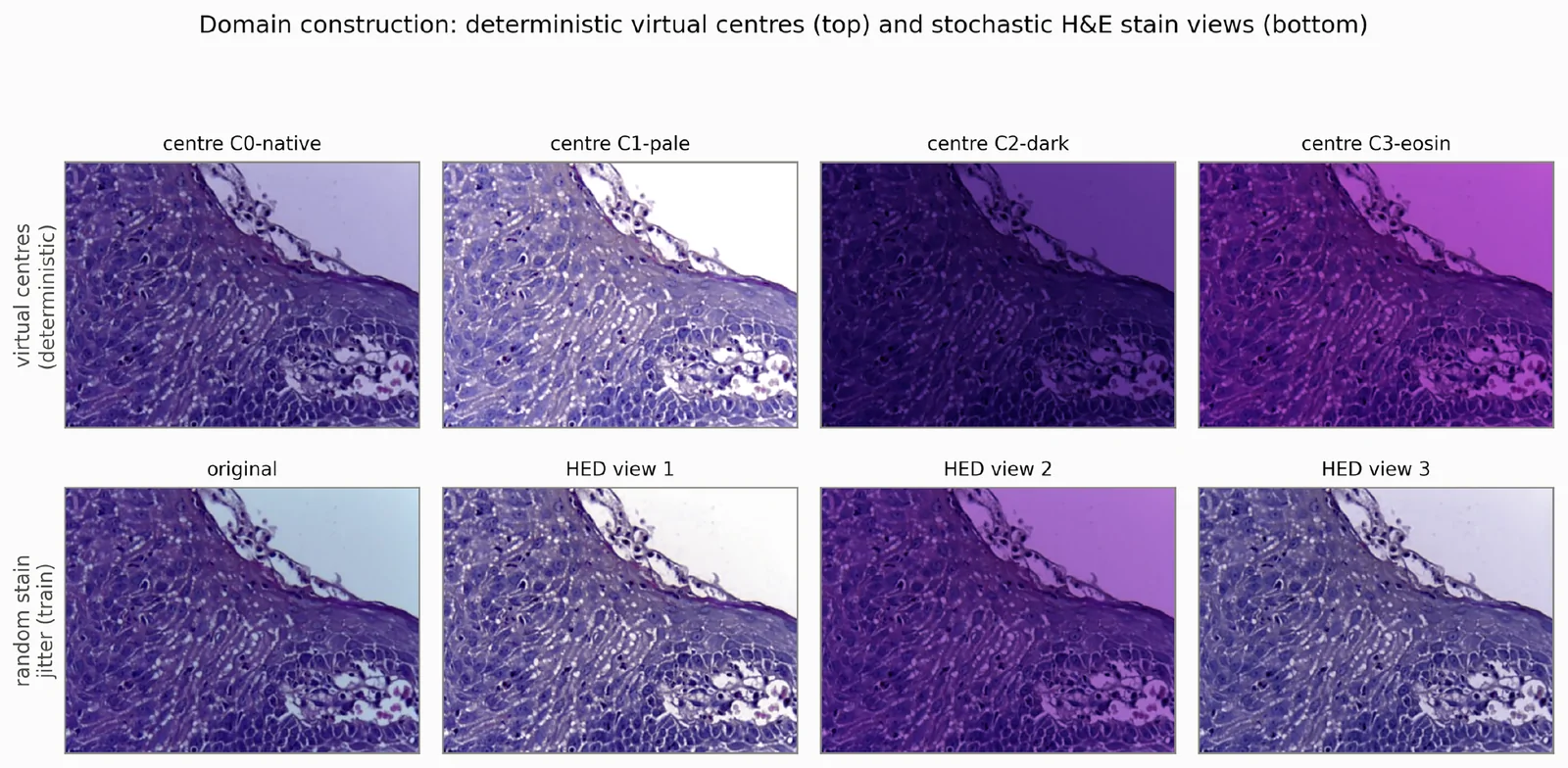
Domain construction and view generation on one image. *Top:* the deterministic per-centre stain profiles that define the virtual centres of the simulated multi-centre protocol. *Bottom:* three independent draws of the training-time HED jitter applied to the same field. Both operators are per-pixel colour maps in haematoxylin–eosin–DAB space, so nuclear morphology, chromatin texture and tissue architecture are identical in every panel; only the H&E appearance differs. This is the paired signal that drives the stain-consistency objective, and the visual counterpart of the structural-similarity measurements reported above. Rahman TY, Mahanta LB, Das AK, Sarma JD. Histopathological imaging database for oral cancer analysis. Data Brief. (2020) 29:105114. doi: 10.1016/j.dib.2020.105114 — dataset deposited as Mendeley Data, doi: 10.17632/ftmp4cvtmb.2. Creative Commons Attribution 4.0 International (CC BY 4.0), as stated on the Mendeley Data record. Mendeley Data, https://data.mendeley.com/datasets/ftmp4cvtmb/2. The panels are our own renderings (colour transforms, crops, annotation) generated from the deposited image files.

Figure 2 shows what the two transforms do to one image: the top row is the same field rendered as four different virtual centres, the bottom row three independent training-time jitters of it. Every panel shares the same nuclei, chromatin pattern and tissue architecture; only the colour changes. Equation 2 is a per-pixel colour map: it rescales stain concentrations and brightness but moves, adds and removes nothing, so nuclear morphology, chromatin texture and tissue architecture are mathematically invariant. The only empirical deviation comes from the finite precision of the colour-deconvolution round trip and 8-bit quantisation. Measured on 120 images per centre by comparing the luminance-standardised original with its recoloured version, the identity centre C0-native—which recolours nothing and therefore isolates the round-trip error alone—attains a structural similarity of 0.989 and a gradient-magnitude correlation of 0.990, and the three strong recolourings C2-dark, C3-eosin and C4-hema attain 0.986–0.989 and 0.987–0.990, indistinguishable from that numerical floor. Only the extreme low-contrast centre C1-pale falls below it (0.910 and 0.884), as expected when the dynamic range is compressed towards white. A complementary check addresses the concern that the transform might create artificial class cues: a logistic probe restricted to *colour statistics only* (37 features, no spatial information) is no better at separating OSCC from normal inside a recoloured centre than inside the native one (Section 4.11).

### 3.5 Data provenance, duplicate audit and leakage control

All experiments use the public *Histopathological imaging database for oral cancer analysis* (27): 1,224 H&E images (100×: 89 normal, 439 OSCC; 400×: 201 normal, 495 OSCC) captured with a Leica DM750 microscope and ICC50 HD camera from biopsies of 230 patients at two Indian institutions. Two facts govern what can be claimed about leakage. First, 1,224 images from 230 patients is 5.3 images per patient, so several images per patient—and very probably per slide—are arithmetically certain. Second, the archive contains images only: no patient, specimen, slide or capture identifier, no accompanying table and no usable acquisition metadata, so *patient-level splitting is impossible with the public data and we do not claim it*. What is possible is to control the leakage channel detectable from the pixels, which we do in three stages, released as scripts/leakage_audit.py. The concern is well founded: tiles of the same subject appearing on both sides of a split inflate reported scores in digital pathology by a wide margin (85), and leakage of this kind is among the most common causes of irreproducible machine-learning results in the sciences (86, 87).

#### 3.5.1 Stage 1: exact duplicates

**Figure 3:**
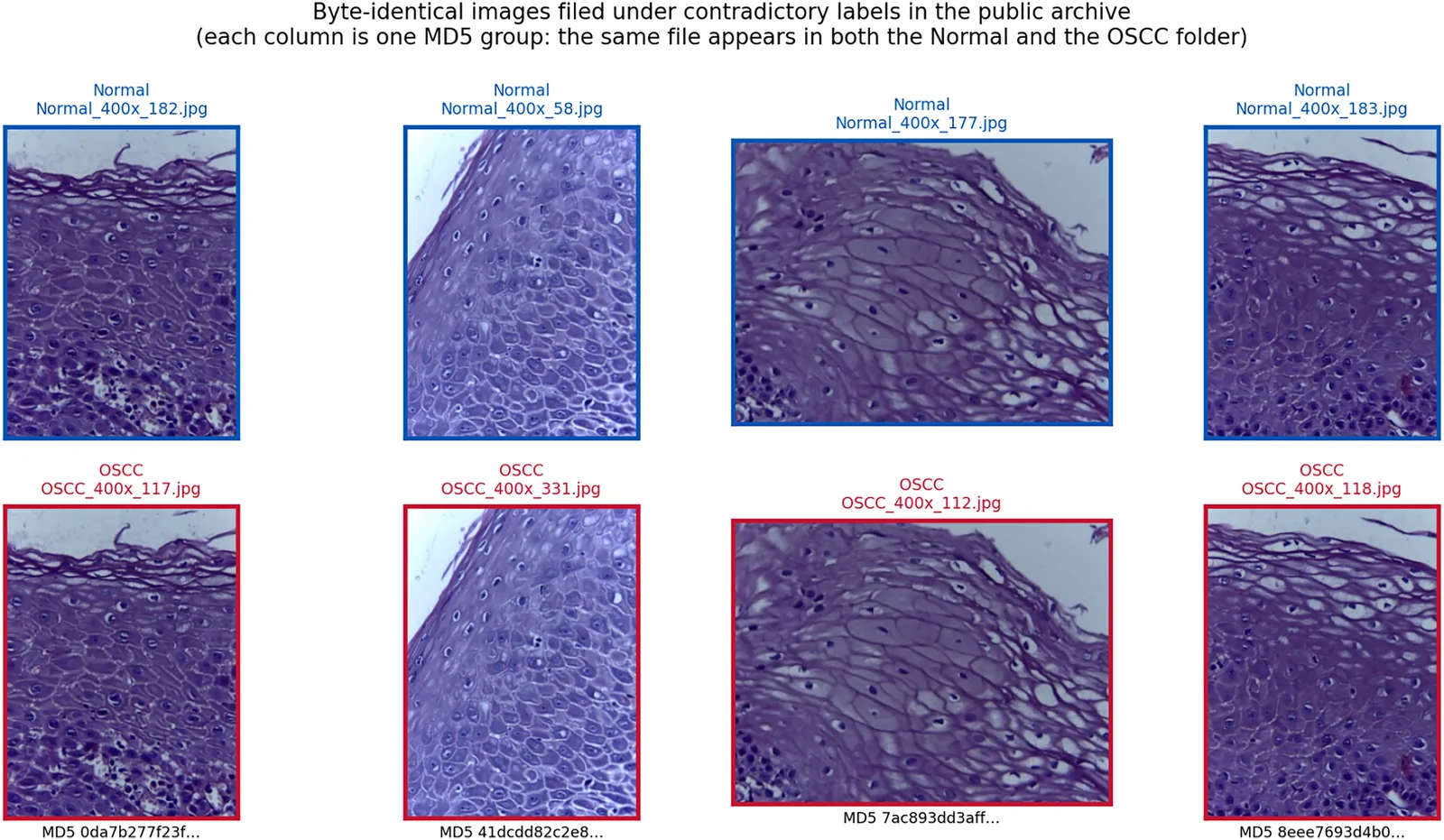
Four of the fourteen groups of byte-identical images that the public archive files under *both* class labels. Each column is one MD5 group: the upper image is the copy stored in the Normal folder and the lower image the copy stored in the OSCC folder; the two files are identical to the byte, so the two labels cannot both be correct. In total 28 images (2.3% of the corpus) are affected, 14 of them from the normal class (4.8% of it). All 16 duplicate groups, their digests and the de-duplicated manifest are released with the code. Rahman TY, Mahanta LB, Das AK, Sarma JD. Histopathological imaging database for oral cancer analysis. Data Brief. (2020) 29:105114. doi: 10.1016/j.dib.2020.105114 — dataset deposited as Mendeley Data, doi: 10.17632/ftmp4cvtmb.2. Creative Commons Attribution 4.0 International (CC BY 4.0), as stated on the Mendeley Data record. Mendeley Data, https://data.mendeley.com/datasets/ftmp4cvtmb/2. The panels are our own renderings (colour transforms, crops, annotation) generated from the deposited image files.

Hashing the raw bytes of all 1,224 files yields only 1,208 distinct MD5 digests. Sixteen groups contain byte-identical files, and in *fourteen* the identical image is filed under *both* class folders—for example Normal_400x_176–183 are bit-for-bit identical to OSCC_400x_111–118, and Normal_100x_72–74 to OSCC_100x_345–347. The contiguous, parallel index runs are the signature of a block of images from one slide having been copied into both class folders when the archive was assembled; Figure 3 shows four of the fourteen. In total 28 images (2.3% of the corpus) carry mutually contradictory labels—14 normal images, i.e. 4.8% of the normal class, each paired with an identical file labelled OSCC—and two further pairs are redundant within-class copies. To our knowledge this has not been reported before, and it matters beyond our study: any model trained on this dataset is fitted against a small block of irreducibly contradictory supervision, and any split placing the two copies on opposite sides leaks a test image into training with the opposite label.

#### 3.5.2 Stage 2: near-duplicates

Beyond byte equality we link two images when a 64-bit difference hash of their grey thumbnails differs in at most 6 bits *or* their ImageNet ResNet-18 embeddings have cosine similarity at least 0.98; the transitive closure of these links defines a group. Both thresholds sit far in the tail of the empirical distribution over all 748,476 pairs (median cosine 0.709, 99th percentile 0.892, 99.9th percentile 0.926), and the counts are stable around them (25 pairs at 0.98, 21 at 0.99). The audit finds 31 multi-image groups covering 65 images (5.3%), the largest containing 5; sixteen are the exact duplicates of Stage 1 and fifteen are genuine near-duplicates. Under the random per-image centre assignment of the original protocol, 26 of the 31 groups were split across two or more virtual centres.

#### 3.5.3 Stage 3: the leakage-controlled protocol

We therefore repeat the multi-centre benchmark on a corrected corpus. All 28 label-contradicted images are removed—their true label is unrecoverable—and each within-class duplicate is collapsed to a single copy, leaving 1,194 images (275 normal, 919 OSCC). Virtual centres are assigned *by group* rather than by image, with greedy load balancing inside every (class × magnification) stratum, and the source train/validation split is likewise group-aware, so that no near-duplicate group is split between a training and a held-out domain (verified: zero spanning groups, against 26 before). Section 4.10 reports how much the results change. Residual same-patient overlap cannot be excluded, because the same slide collection was imaged at both magnifications and no identifiers were released; this irreducible limitation is stated in Section 5.

### 3.6 Feature extractor with MixStyle style randomization

The backbone $F_{\theta }$ is an ImageNet-pretrained ResNet-18 that is shared, with tied weights, across the two stain views in a Siamese manner, ensuring that both colour variants are pushed through an identical mapping. To preserve generic low-level filters and stabilise optimisation on a moderately sized dataset, the stem and the first residual stage are frozen while the deeper stages are fine-tuned, and a global average pooling produces a compact feature $f\in R^{d}$ with d=512. To discourage the network from using scanner- or centre-specific *style*—encoded largely in channel-wise feature statistics—as a discriminative shortcut, MixStyle modules (18, 54) are inserted between the early residual stages. The operator below is reproduced without modification; it is the stochastic counterpart of adaptive instance normalisation (57), which replaces the channel statistics of a content feature map by those of a style image, whereas MixStyle replaces them by a convex combination of the statistics of two training samples. For an intermediate feature map $u\in R^{C\times H\times W}$, MixStyle first computes the instance-level channel mean and standard deviation given in Equation 3,

$$\mu (u{)}_{c}=\frac{1}{HW}\sum_{h=1}^{H}\sum_{w=1}^{W}u_{c,h,w},\;\sigma (u{)}_{c}=\sqrt{\frac{1}{HW}\sum_{h=1}^{H}\sum_{w=1}^{W}{\left(u_{c,h,w}-\mu (u{)}_{c}\right)}^{2}+\epsilon }\;,$$ (3)

and then, for a sample $u_{i}$ and a randomly permuted partner $u_{\pi (i)}$ in the mini-batch, mixes their statistics with a coefficient λ∼Beta(a,a) (53) before re-normalising the content of $u_{i}$ according to Equation 4:

$$\mathrm{MixStyle}(u_{i})=\underset{\sigma_{\mathrm{mix}}}{\underbrace{\left(\lambda \;\sigma (u_{i})+(1-\lambda )\;\sigma (u_{\pi (i)})\right)}}\cdot \frac{u_{i}-\mu (u_{i})}{\sigma (u_{i})}+\underset{\mu_{\mathrm{mix}}}{\underbrace{\left(\lambda \;\mu (u_{i})+(1-\lambda )\;\mu (u_{\pi (i)})\right)}}.$$ (4)

The mixing statistics are treated as constants with respect to the gradient, the operation is applied stochastically with probability p and is disabled at inference, so that MixStyle acts purely as a parameter-free, training-time style-augmentation that synthesises novel appearances along the haematoxylin/eosin axes without altering the underlying tissue content.

### 3.7 Stain-consistency regularization

The first explicit invariance objective forces the two stain views of every image to be encoded consistently, thereby making the representation robust to H&E colour while retaining discriminative structure. Let $f^{\left(v\right)}=F_{\theta }({\tilde{x}}^{\left(v\right)})$ and $\ell^{\left(v\right)}=C_{\phi }(f^{\left(v\right)})$ denote the feature and logits of view v∈{1,2}, and let $z^{\left(v\right)}=g_{\psi }(f^{\left(v\right)})/\|g_{\psi }(f^{\left(v\right)}){\|}_{2}$ be the $\ell_{2}$-normalised output of a non-linear projection head $g_{\psi }$. Consistency is imposed in the embedding space through a symmetric negative-cosine objective (60) with a stop-gradient on the target branch in the form used by SimSiam (61),

$$L_{\mathrm{emb}}=\frac{1}{2}\left[\left(2-2\;\langle z^{\left(1\right)},\;\mathrm{sg}[z^{\left(2\right)}]\rangle \right)+\left(2-2\;\langle z^{\left(2\right)},\;\mathrm{sg}[z^{\left(1\right)}]\rangle \right)\right],$$ (5)

where ⟨⋅,⋅⟩ is the inner product and sg⁡[⋅] denotes the stop-gradient operator. We deliberately state what this operator does and does not do here, since SICA adopts the SimSiam objective but not its asymmetric predictor head. Because Equation 5 is *symmetric*, the two stop-gradients cancel: its gradient with respect to the parameters is exactly $-\nabla_{\theta ,\psi }\langle z^{\left(1\right)},z^{\left(2\right)}\rangle$, i.e. parallel to that of the plain cosine objective and equal to it up to the factor 1/2 (we verified this numerically; the gradient cosine is 1.000). The stop-gradient is therefore an implementation convenience in our setting, and it is *not* what prevents a degenerate solution. What prevents it is that the projector is an auxiliary branch on features that are simultaneously trained by the class-weighted cross-entropy of Equation 10: a constant embedding would destroy the discriminative signal the classification term requires. We make no claim to inherit SimSiam’s collapse-avoidance guarantee, which is established for the asymmetric predictor configuration. Consistency is simultaneously enforced in the prediction space by matching the two temperature-softened class distributions $p^{\left(v\right)}=\mathrm{softmax}(\ell^{\left(v\right)}/T)$ through a symmetric, temperature-rescaled Kullback–Leibler divergence given in Equation 6,

$$L_{\mathrm{pred}}=\frac{T^{2}}{2}\left[\mathrm{KL}\left(\mathrm{sg}[p^{\left(2\right)}]\;\|\;p^{\left(1\right)}\right)+\mathrm{KL}\left(\mathrm{sg}[p^{\left(1\right)}]\;\|\;p^{\left(2\right)}\right)\right],$$ (6)

in which the temperature T softens the targets and the $T^{2}$ factor keeps the gradient magnitude comparable to that of the classification term [both devices are taken from knowledge distillation (62), with the two views acting as each other’s teacher, and the symmetrisation follows deep mutual learning (75, 76)]. The overall stain-consistency loss of Equation 7 is the sum of the two complementary terms,

$$L_{\mathrm{cons}}=L_{\mathrm{emb}}+L_{\mathrm{pred}},$$ (7)

and because both views share identical geometry and content, minimising $L_{\mathrm{cons}}$ drives the model to become invariant specifically to the H&E colour perturbation rather than to any semantic factor.

### 3.8 Class-conditional feature alignment

The second invariance objective removes residual domain-specific offsets by aligning the source-domain feature distributions, but does so *within each class* so as not to blur the boundary between normal and malignant tissue. For a pair of feature sets A and B, we adopt a discrepancy that matches both the first- and the second-order statistics,

$$\ell_{\mathrm{CORAL}}(A,B)=\frac{1}{d}\;{\left\|\mu_{A}-\mu_{B}\right\|}_{2}^{2}+\frac{1}{4d^{2}}\;{\left\|\Sigma_{A}-\Sigma_{B}\right\|}_{F}^{2},$$ (8)

where $\mu_{A}$ and $\Sigma_{A}$ are the empirical mean vector and covariance matrix of A, $\|\cdot {\|}_{F}$ is the Frobenius norm, and d is the feature dimension. The second term, including its $1/(4d^{2})$ normalisation, is the Deep CORAL loss (48, 77) used unchanged; adding the first-order term makes Equation 8 an order-two central moment discrepancy (78, 79) rather than CORAL proper, and we name it accordingly. Restricting such an alignment to matched class-conditional distributions is likewise an established device (80–83); what is specific to SICA is the instantiation—class-conditional CORAL averaged over every valid (class, domain-pair) triple inside a class-balanced mini-batch, combined with the two view-based invariances above. Let $F_{k,c}=\{\;f_{i}\;:\;d_{i}=k,\;y_{i}=c\;\}$ collect the first-view features of class c that originate from source domain k, and let P be the set of all valid $\left(c,k,k^{\prime }\right)$ triples for which both groups contain at least two samples. The class-conditional alignment loss then averages the pairwise discrepancy over classes and over every ordered pair of source domains,

$$L_{\mathrm{align}}=\frac{1}{\left|P\right|}\sum_{c\in Y}\;\sum_{\begin{array}{c} k<k^{\prime }\\ (c,k,k^{\prime })\in P \end{array}}\ell_{\mathrm{CORAL}}\left(F_{k,c},\;F_{k^{\prime },c}\right).$$ (9)

By conditioning on the label, Equation 9 suppresses domain-induced covariate shift while preserving the discriminative geometry that a purely marginal alignment would tend to collapse, and a class-agnostic variant—in which the class index is dropped and features are pooled per domain—is retained only as an ablation. Together with the consistency term of Section 3.7 and the MixStyle randomisation of Section 3.6, this component completes the three complementary invariances that constitute the proposed method.

### 3.9 Overall training objective and optimization

The supervised signal is a class-weighted cross-entropy averaged over the two stain views, which encourages a correct prediction irrespective of the sampled colour,

$$L_{\mathrm{cls}}=\frac{1}{2}\sum_{v\in \{1,2\}}\mathrm{CE}\left(\ell^{\left(v\right)},\;y\right),\;\mathrm{CE}(\ell ,y)=-\sum_{c\in Y}w_{c}\;1[y=c]\;\log\mathrm{softmax}(\ell {)}_{c},$$ (10)

where $w_{c}$ are optional per-class weights and 1[⋅] is the indicator function. The three objectives are combined into a single loss in which the two regularisers are gradually introduced through a linear warm-up schedule $\lambda (t)=\min\;(1,\;t/T_{w})$, with t the current step and $T_{w}$ the warm-up length, so that a reliable feature space is established before invariance is enforced,

$$L=L_{\mathrm{cls}}+\lambda (t)\left(w_{\mathrm{cons}}\;L_{\mathrm{cons}}+w_{\mathrm{align}}\;L_{\mathrm{align}}\right).$$ (11)

Setting either weight to zero, or emptying the MixStyle layers, recovers the corresponding ablation, so the formulation degrades gracefully to each of its components. Because the dataset is markedly imbalanced towards OSCC, class balance is additionally enforced at the sampling level: within each source domain, sample i is drawn with probability proportional to $1/n_{y_{i}}$, where $n_{c}$ is the number of training images of class c in that domain, and one balanced mini-batch is drawn per source domain at every optimisation step. The whole model is trained end-to-end with the Adam optimiser using a fixed learning rate and weight decay, which, together with Equation 11, jointly optimises the featurizer, the classifier, and the projection head.

### 3.10 Evaluation protocol and metrics

Model selection follows the standard training-domain validation protocol for domain generalization, in which a stratified 20% slice of the *source* domains—never the target—is held out and the checkpoint attaining the best validation area under the receiver-operating-characteristic curve (AUROC) is retained. The selected model is then evaluated once on the untouched target domain, and, to characterise performance under class imbalance, we report a panel of complementary metrics rather than accuracy alone. Denoting by TP,TN,FP,FN the confusion-matrix counts with OSCC as the positive class, we report sensitivity (OSCC recall) and specificity (normal recall) together with their balanced average, as defined in Equation 12,

$$\mathrm{Sen}=\frac{\mathrm{TP}}{\mathrm{TP}+\mathrm{FN}},\;\mathrm{Spe}=\frac{\mathrm{TN}}{\mathrm{TN}+\mathrm{FP}},\;\mathrm{BAcc}=\frac{1}{2}\left(\mathrm{Sen}+\mathrm{Spe}\right),$$ (12)

as well as the $F_{1}$ score of the positive class and the Matthews correlation coefficient, which is informative for skewed distributions, both given in Equation 13,

$$F_{1}=\frac{2\;\mathrm{TP}}{2\;\mathrm{TP}+\mathrm{FP}+\mathrm{FN}},\;\mathrm{MCC}=\frac{\mathrm{TP}\cdot \mathrm{TN}-\mathrm{FP}\cdot \mathrm{FN}}{\sqrt{\left(\mathrm{TP}+\mathrm{FP})(\mathrm{TP}+\mathrm{FN})(\mathrm{TN}+\mathrm{FP})(\mathrm{TN}+\mathrm{FN}\right)}}.$$ (13)

In addition, we summarise the threshold-independent ranking quality with the AUROC and the area under the precision–recall curve (AUPRC), the latter being particularly sensitive to the minority behaviour of the positive class. All scores are computed under the LODO protocol and aggregated as the mean and standard deviation over the leave-one-domain-out folds and multiple random seeds, and the proposed method is benchmarked against nine established domain-generalization baselines under an identical training and evaluation pipeline.

#### 3.10.1 Calibration

Because a screening score is useful only if its probability can be trusted at a new centre, we also quantify calibration. With the held-out images partitioned into B bins $I_{1},\ldots ,I_{B}$ of equal *mass* (equal count, which avoids the empty high-confidence bins that equal-width binning produces under class imbalance), and ${\bar{p}}_{b}$, ${\bar{y}}_{b}$ the mean predicted probability and observed OSCC rate in bin b, we report the expected and maximum calibration error of Equation 14 (88, 89)

$$\mathrm{ECE}=\sum_{b=1}^{B}\frac{\left|I_{b}\right|}{N}\left|{\bar{y}}_{b}-{\bar{p}}_{b}\right|,\;\mathrm{MCE}=\max_{b}\left|{\bar{y}}_{b}-{\bar{p}}_{b}\right|,$$ (14)

with B=15, the Brier score (90) $\mathrm{BS}=\frac{1}{N}\sum_{i}(p_{i}-y_{i}{)}^{2}$ with its reliability/resolution/uncertainty decomposition, and—because ECE depends on the binning—the calibration slope $\hat{b}$ and calibration-in-the-large $\hat{a}$ (91) from the bin-free logistic recalibrations

$$\mathrm{Pr}(y\;=\;1)=\sigma \;\left(a_{0}+b\;\mathrm{logit}(p)\right),\;\mathrm{Pr}(y\;=\;1)=\sigma \;\left(a+\mathrm{logit}(p)\right),$$ (15)

where σ is the logistic function. A perfectly calibrated model has $\hat{b}=1$, $\hat{a}=0$; $\hat{b}<1$ indicates over-confident probabilities. Calibration is computed per held-out fold and then averaged, since pooling folds of differing prevalence lets over- and under-estimation cancel.

#### 3.10.2 Statistical analysis

Every aggregate is accompanied by a 95% confidence interval computed across the D×S leave-one-domain-out runs (D held-out domains, S=3 seeds) with Student’s t quantiles, and per-domain AUROC is additionally given percentile bootstrap intervals (92) over 2,000 stratified resamples of the held-out images. For the comparison of SICA with each baseline we exploit the fact that the two are evaluated on *identical* held-out images in every fold and seed, and use paired tests. As the primary test we average the predicted probabilities over seeds within each held-out domain, apply DeLong’s test for two correlated ROC curves (93) to obtain one difference and standard error per domain, and pool the D estimates with a DerSimonian–Laird random-effects model, which propagates the between-domain heterogeneity a fixed-effect pooling would ignore. As a secondary, distribution-free test we use an exact two-sided sign-flip permutation test on the D×S paired run-level differences, cross-checked with the Wilcoxon signed-rank test, reporting the matched-pairs rank-biserial correlation as effect size. All p-values are corrected across the nine baselines within a protocol by Holm–Bonferroni (94), following standard practice for comparing multiple classifiers on a benchmark (95). Two properties bound what any experiment on this benchmark can establish: with n paired runs the smallest attainable two-sided permutation p-value is $2^{1-n}$, which is $6.1\times 10^{-5}$ for the multi-centre protocol (n=15) but 0.031 for cross-magnification (n=6), so after correction for nine comparisons no confirmatory claim is attainable on the latter; and the three seeds within a fold share a test set, so the effective number of independent units for a domain-level claim is D rather than D×S. We therefore treat the multi-centre protocol as confirmatory and cross-magnification as supportive, reporting effect sizes with intervals there rather than significance.

## 4 Experimental results

### 4.1 Evaluation protocols, baselines and metrics

We evaluate every method under the two leave-one-domain-out (LODO) protocols of Section 3: a controlled *simulated multi-centre* shift, in which the pooled images are recoloured into K=5 virtual centres (C0-native, C1-pale, C2-dark, C3-eosin, C4-hema) differing only in H&E appearance, and a genuine *cross-magnification* shift, in which 100× and 400× images form two single-source domains. The former isolates stain shift as a multi-source problem, the latter is a hard single-source problem confounding scale, field of view and colour. SICA is compared against nine baselines spanning the main families: empirical risk minimisation (ERM), classical stain normalisation (Macenko-Norm), adversarial feature learning (DANN), moment- and kernel-matching alignment (Deep CORAL, MMD), invariant-risk and worst-case objectives (IRM, GroupDRO), and augmentation-based regularisers (inter-domain Mixup, MixStyle). All share the same ImageNet-pretrained ResNet-18 backbone, class-balanced sampling, training budget and *training-domain* model-selection rule, so differences reflect the generalization principle rather than the architecture. Three further baselines were added in revision to complete the stain-handling families—Reinhard and Vahadane normalisation and a strong stain-augmentation regime (Section 4.11)—and frozen pathology-foundation-model encoders are compared separately in Section 4.13, where the backbone is deliberately *not* held fixed. Table 1 categorises the methods by mechanism, by the level at which they act and by whether their objective requires multiple source domains, which explains their behaviour in the single-source protocol. Unless stated otherwise, every entry is mean ± standard deviation over the held-out folds and three random seeds, with sensitivity defined as OSCC recall and specificity as Normal recall.

**Table 1:** Taxonomy of the evaluated methods. “Level” is where the mechanism acts; “≥2 sources” indicates whether the objective requires multiple source domains, so that methods marked ✓degenerate to ERM in the single-source cross-magnification protocol (Section 4.4); “Stain views” indicates use of the paired H&E views.

Method	Family	Invariance/regularisation principle	Level	≥2 sources	Stain views
ERM	Risk minimisation	None (empirical risk, with stain augmentation)	Loss	No	No
Macenko-Norm	Stain normalisation	Canonicalise H&E colour to a reference	Input	No	Normalises
Reinhard-Norm	Stain normalisation	Match LAB channel statistics to a reference	Input	No	Normalises
Vahadane-Norm	Stain normalisation	Sparse NMF stain separation, re-project on a reference	Input	No	Normalises
Strong stain aug.	Augmentation	Heavy HED colour jitter on every training image	Input	No	No
DANN	Adversarial	Domain-adversarial (indistinguishable) features	Feature	✓	No
Deep CORAL	Moment matching	Align second-order feature statistics across domains	Feature	✓	No
MMD	Kernel matching	Align feature distributions via multi-kernel MMD	Feature	✓	No
IRM	Invariant prediction	Predictor optimal across all environments	Loss	✓	No
GroupDRO	Distributional robust.	Minimise the worst-case source-domain risk	Loss	✓	No
Mixup	Augmentation	Convex interpolation of cross-domain samples	Input	✓	No
MixStyle	Augmentation	Mix channel-wise feature statistics (style)	Feature	No	No
SICA (ours)	Consistency + align.	Style randomisation + stain-consistency + class-conditional alignment	Feature + loss	No	✓

The three red rows were added in revision to complete the stain-normalisation and stain-augmentation families (Section 4.11); frozen foundation-model encoders are compared separately in Section 4.13 because they change the backbone rather than the objective.

### 4.2 Overall comparison

**Figure 4:**
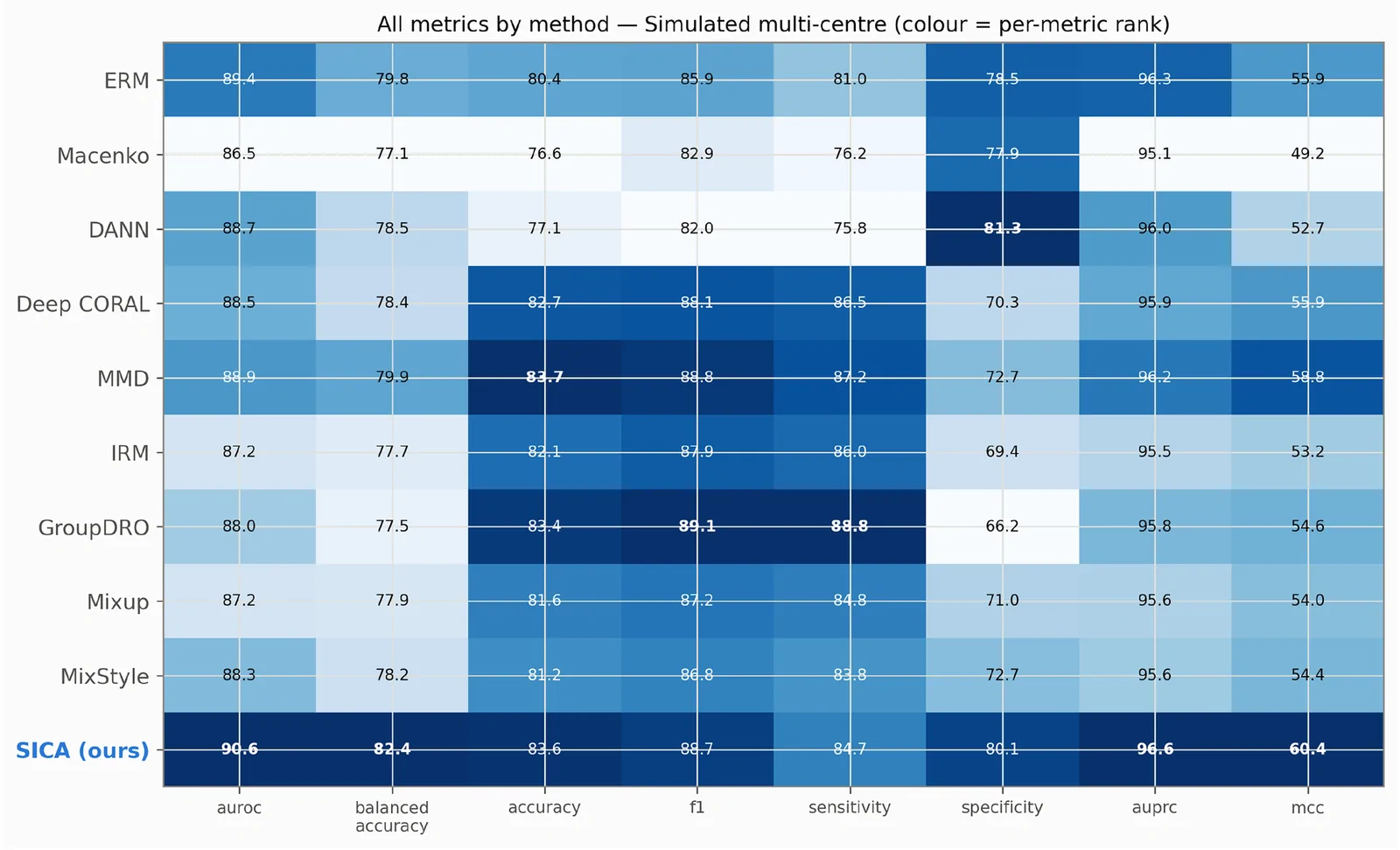
All eight metrics for every method on the simulated multi-centre protocol; colour encodes the per-metric rank (darker is better). SICA occupies the best or near-best cell in the discrimination and correlation columns while remaining balanced across sensitivity and specificity. The best value per metric is in **bold**; the proposed method is identified by the colour of its row label.

Table 2 reports all eight metrics for every method under both protocols. The gains of SICA over the ERM reference are positive on every metric of the multi-centre protocol and on all but specificity of the cross-magnification protocol. On the realistic multi-centre benchmark SICA attains the best mean AUROC of 90.6% among the methods sharing the common ResNet-18 backbone (frozen foundation-model encoders are compared separately in Section 4.13), exceeding ERM by 1.15 percentage points (pp) and the strongest baseline, MMD (88.9%), by 1.63 pp, while simultaneously achieving the *smallest* standard deviation (±2.2) of any method, i.e. it is both the most accurate and the most stable. Beyond ranking quality, SICA also obtains the highest balanced accuracy (82.4%), AUPRC (96.6%) and Matthews correlation coefficient (60.4), the last being a 1.6-point margin over the best baseline (MMD, 58.8) and a metric that is particularly informative under the strong OSCC/Normal imbalance. A closer look at the class-wise recalls reveals *why* SICA is preferable in a screening context: the alignment- and robustness-oriented baselines (Deep CORAL, MMD, IRM, GroupDRO) push OSCC sensitivity up to 86%–89% but at the cost of Normal specificity collapsing to 66%–73%, meaning they systematically over-predict cancer, whereas Macenko and DANN trade in the opposite direction; SICA instead delivers a well-balanced operating point (84.7% sensitivity and 80.1% specificity) that neither over- nor under-calls OSCC, and this balance holds across the full metric panel. The per-metric rank map in Figure 4 makes the pattern explicit: SICA occupies the top-ranked cells for the discrimination and correlation metrics while remaining competitive on the others. On the far harder cross-magnification protocol the absolute numbers drop into a near-chance regime for most baselines, yet SICA still leads with 65.7% AUROC vs. 61.6% for ERM (+4.0 pp) and is best on seven of the eight metrics, indicating that the advantage is not an artefact of the easier synthetic shift.

**Table 2:** Domain-generalization results under both LODO protocols.

		Discrimination		Balance		Overall		Class-wise recall	
Protocol	Method	AUROC	AUPRC	Bal. Acc	MCC	Accuracy	F1	Sensitivity	Specificity
Simulated multi-centre (K=5)	ERM	89.4 ± 2.4	96.3 ± 0.8	79.8 ± 5.9	55.9 ± 9.5	80.4 ± 7.3	85.9 ± 6.8	81.0 ± 12.3	78.5 ± 16.4
	Macenko-Norm	86.5 ± 4.2	95.1 ± 2.1	77.1 ± 3.8	49.2 ± 6.6	76.6 ± 5.9	82.9 ± 5.7	76.2 ± 10.8	77.9 ± 13.4
	DANN	88.7 ± 4.8	96.0 ± 1.7	78.5 ± 7.6	52.7 ± 13.5	77.1 ± 12.7	82.0 ± 14.4	75.8 ± 18.4	**81.3 ± 11.8**
	Deep CORAL	88.5 ± 4.3	95.9 ± 1.7	78.4 ± 5.2	55.9 ± 10.0	82.7 ± 6.0	88.1 ± 5.4	86.5 ± 9.6	70.3 ± 12.7
	MMD	88.9 ± 3.9	96.2 ± 1.4	79.9 ± 4.4	58.8 ± 9.5	**83.7 ± 6.1**	88.8 ± 5.3	87.2 ± 9.5	72.7 ± 10.1
	IRM	87.2 ± 4.6	95.5 ± 1.7	77.7 ± 7.1	53.2 ± 11.9	82.1 ± 4.2	87.9 ± 3.1	86.0 ± 5.7	69.4 ± 15.9
	GroupDRO	88.0 ± 6.0	95.8 ± 2.1	77.5 ± 8.9	54.6 ± 15.6	83.4 ± 4.8	**89.1 ± 3.3**	**88.8 ± 5.8**	66.2 ± 19.4
	Mixup	87.2 ± 6.2	95.6 ± 2.1	77.9 ± 7.6	54.0 ± 14.1	81.6 ± 7.7	87.2 ± 6.5	84.8 ± 10.4	71.0 ± 14.4
	MixStyle	88.3 ± 3.3	95.6 ± 1.4	78.2 ± 4.7	54.4 ± 7.0	81.2 ± 5.4	86.8 ± 5.0	83.8±10.4	72.7 ± 15.6
	SICA (ours)	**90.6 ± 2.2**	**96.6 ± 1.1**	**82.4 ± 2.4**	**60.4 ± 4.6**	83.6±3.1	88.7 ± 2.6	84.7 ± 5.6	80.1 ± 7.8
Cross-magnification	ERM	61.6 ± 9.0	84.0 ± 4.6	57.7 ± 5.8	13.5 ± 11.2	52.9 ± 19.1	58.9 ± 24.9	51.6 ± 27.3	63.8 ± 18.2
	Macenko-Norm	55.4 ± 10.1	80.5 ± 9.6	51.1 ± 5.3	1.7 ± 9.7	54.1 ± 12.8	64.0 ± 16.5	58.1 ± 20.9	44.0 ± 25.6
	DANN	58.6 ± 6.7	82.8 ± 5.6	54.2 ± 5.4	7.6 ± 8.3	48.7 ± 10.6	55.9 ± 12.9	45.7 ± 21.5	62.6 ± 23.5
	Deep CORAL	61.6 ± 9.0	84.0 ± 4.6	57.7 ± 5.8	13.5 ± 11.2	52.9 ± 19.1	58.9 ± 24.9	51.6 ± 27.3	63.8 ± 18.2
	MMD	61.6 ± 9.0	84.0 ± 4.6	57.7 ± 5.8	13.5 ± 11.2	52.9 ± 19.1	58.9 ± 24.9	51.6 ± 27.3	63.8 ± 18.2
	IRM	59.0 ± 12.7	83.3 ± 3.7	55.8 ± 8.0	9.6 ± 17.5	46.7 ± 22.4	49.5 ± 30.7	43.1 ± 32.1	**68.6 ± 19.6**
	GroupDRO	61.6 ± 9.0	84.0 ± 4.6	57.7 ± 5.8	13.5 ± 11.2	52.9 ± 19.1	58.9 ± 24.9	51.6±27.3	63.8 ± 18.2
	Mixup	56.3 ± 13.9	82.1 ± 4.1	55.8 ± 7.4	10.3 ± 14.2	47.2 ± 21.0	51.1 ± 28.4	44.8 ± 32.1	66.8 ± 25.8
	MixStyle	64.5 ± 4.2	83.9 ± 5.8	58.0 ± 4.3	15.6 ± 8.9	59.0 ± 17.5	66.6 ± 22.5	62.3 ± 27.9	53.7 ± 21.0
	SICA (ours)	**65.7 ± 8.1**	**85.4 ± 5.1**	**60.4 ± 4.4**	**19.2 ± 7.9**	**61.4 ± 13.5**	**70.2 ± 13.7**	**63.9 ± 21.4**	56.8 ± 22.5

The upper block is the simulated multi-centre protocol (K=5 virtual stain centres, five leave-one-centre-out folds); the lower block is the cross-magnification protocol (100× vs. 400×, two single-source folds). Both are also named in the row labels at the left edge. Values are mean ± std (%) over held-out domains and three seeds; standard deviations use the unbiased (n−1) estimator throughout the paper; the best result in each column is in **bold**. Metrics are grouped as discrimination (AUROC, AUPRC), balance/correlation (balanced accuracy, MCC), overall (accuracy, F1) and class-wise recall (sensitivity = OSCC recall, specificity = Normal recall).

### 4.3 Per-domain robustness and worst-case behaviour

A benchmark average can hide catastrophic failures on individual centres, so Table 3 reports the held-out AUROC for every domain together with each method’s per-protocol mean and, crucially, its *worst* domain. The hardest held-out centre for essentially every method is C1-pale—the low-contrast, faint-staining centre—and this is where the baselines break down: Mixup, GroupDRO, IRM and Deep CORAL fall to 76.6%, 78.3%, 80.1% and 81.0%, and even ERM drops to 86.9%. SICA sustains 89.2%, a +2.3 pp margin over ERM and a +6–11 pp margin over the alignment methods, so its *worst-case* domain exceeds the *average* of most competitors—the clearest evidence that the stain-consistency objective targets the dominant failure mode. This is not achieved by sacrificing the easy centres: SICA is also best on C0-native (93.8%) and within 0.6 pp of the best on C3-eosin and C4-hema, trailing appreciably only on C2-dark (91.2 against 93.7 for DANN), the centre whose high-contrast stain is least ambiguous; its per-domain spread is the tightest of all methods (89.2%–93.8%). The per-domain differences confirm the pattern: SICA gains on four of the five centres (+2.7, +2.3, +0.4, +1.5 pp on C0/C1/C3/C4) and regresses only on C2-dark (−1.1 pp), where the high-contrast stain already provides a strong signal that style-mixing and consistency can slightly over-regularise. The distribution of SICA’s out-of-domain AUROCs is likewise shifted upward and free of the long left tails that drag Mixup, GroupDRO, IRM and Macenko down toward 73%–77%, so it is both higher on average and far less prone to worst-case collapse.

**Table 3:** Per-held-out-domain AUROC (%) for both protocols, with each method’s mean and worst-case (minimum) domain.

	Simulated multi-centre — held-out centre							Cross-magnification			
Method	C0-native	C1-pale	C2-dark	C3-eosin	C4-hema	Mean	Worst	100×	400×	Mean	Worst
ERM	91.1	86.9	92.4	89.0	87.8	89.4	86.9	57.8	65.5	61.6	57.8
Macenko-Norm	90.0	85.1	91.2	84.4	81.8	86.5	81.8	60.7	50.1	55.4	50.1
DANN	90.5	83.5	**93.7**	86.1	**89.8**	88.7	83.5	55.1	62.2	58.6	55.1
Deep CORAL	91.2	81.0	92.4	**89.5**	88.4	88.5	81.0	57.8	65.5	61.6	57.8
MMD	90.9	83.1	93.6	88.4	88.7	88.9	83.1	57.8	65.5	61.6	57.8
IRM	91.1	80.1	89.9	86.2	88.8	87.2	80.1	49.6	**68.5**	59.0	49.6
GroupDRO	92.7	78.3	92.4	88.1	88.4	88.0	78.3	57.8	65.5	61.6	57.8
Mixup	92.5	76.6	91.2	88.9	87.0	87.2	76.6	45.8	66.7	56.3	45.8
MixStyle	92.6	84.8	89.6	87.7	86.9	88.3	84.8	64.1	65.0	64.5	64.1
SICA (ours)	**93.8**	**89.2**	91.2	89.4	89.3	**90.6**	**89.2**	**64.9**	66.5	**65.7**	**64.9**

Best per column in **bold**. The worst-case column is the key robustness indicator: SICA’s worst domain (C1-pale, 89.2) exceeds the *mean* of most baselines.

### 4.4 Single-source degeneration of alignment baselines

The cross-magnification results in Table 2 expose a structural limitation of the baseline families that is worth isolating, and that is anticipated by the “≥2 sources” column of Table 1. Because this protocol has only a single source magnification, the methods whose loss requires *multiple* source domains—Deep CORAL and MMD (which align statistics across domains), GroupDRO (which reweights domains) and, to a lesser degree, DANN (whose discriminator needs several domain labels)—have no cross-domain signal to exploit and therefore reduce to plain risk minimisation. This is visible directly in the numbers: Deep CORAL, MMD and GroupDRO produce results *numerically identical* to ERM (61.6% AUROC and the same figures on every other metric), because their domain-alignment terms are inactive with a single source.

Because a reviewer rightly flagged the repeated values as a possible sign of duplicated results, we make the mechanism and its verification explicit. Each of these objectives has the form $L=L_{\mathrm{cls}}+\lambda \;R(\{f_{k}{\}}_{k\in S})$, where R is a functional of the features of *several* source domains: Deep CORAL and MMD sum a discrepancy over ordered pairs of distinct domains, and GroupDRO re-weights per-domain risks through a softmax over the domain index. When |S|=1 the pair set is empty and the softmax is degenerate, so R≡0 and ∇R≡0: the three algorithms reduce, term by term, to empirical risk minimisation. Since the benchmark fixes the seed, data order, initialisation and augmentation stream per run, identical objectives give identical trajectories and identical models. We verified this rather than assuming it: hashing the raw predicted probabilities of all 210 benchmark runs shows ERM, Deep CORAL, MMD and GroupDRO to be *bit-identical* in all six cross-magnification runs, with a maximum absolute deviation of exactly 0 over every test image, whereas DANN and SICA differ from ERM by up to 0.92 and 0.80 in predicted probability on the same runs. In the multi-centre protocol, where two or more sources are always active, no two methods share a prediction vector in any of the 15 runs. The repetition is therefore a property of the objectives under single-source training, not a copy-paste artefact, and the audit script that reproduces the check is released with the code. IRM is a partial exception—its penalty is computed per environment and remains non-zero with one source—which is why its numbers differ, as do Mixup’s, whose inter-domain interpolation falls back to within-domain mixing.

### 4.5 ROC, threshold behaviour and the sensitivity–specificity trade-off

The pooled ROC curves on held-out data tell the same story on both protocols: on the multi-centre protocol SICA’s curve dominates across nearly the entire operating range, reaching a pooled AUC of 0.899 against 0.865–0.884 for the competing methods, and on cross-magnification the separation is proportionally larger (0.631 vs. 0.557–0.594). Translated into error counts, relative to ERM SICA reduces false positives (Normal misclassified as OSCC, 187→173) and false negatives (OSCC missed, 531→428) *at the same time*, lifting Normal specificity from 78.5% to 80.1% and OSCC sensitivity from 81.0% to 84.7%; simultaneously improving both error types is unusual, because a single threshold ordinarily trades one against the other. The alignment baselines cluster toward the high-sensitivity/low-specificity corner and Macenko/DANN toward the opposite one, whereas SICA sits at the favourable upper-right region, dominating most baselines in the Pareto sense. Such a balanced operating point is what an unsupervised-target screening tool requires, since both missed cancers and unnecessary referrals carry clinical cost.

### 4.6 Calibration

**Figure 5:**
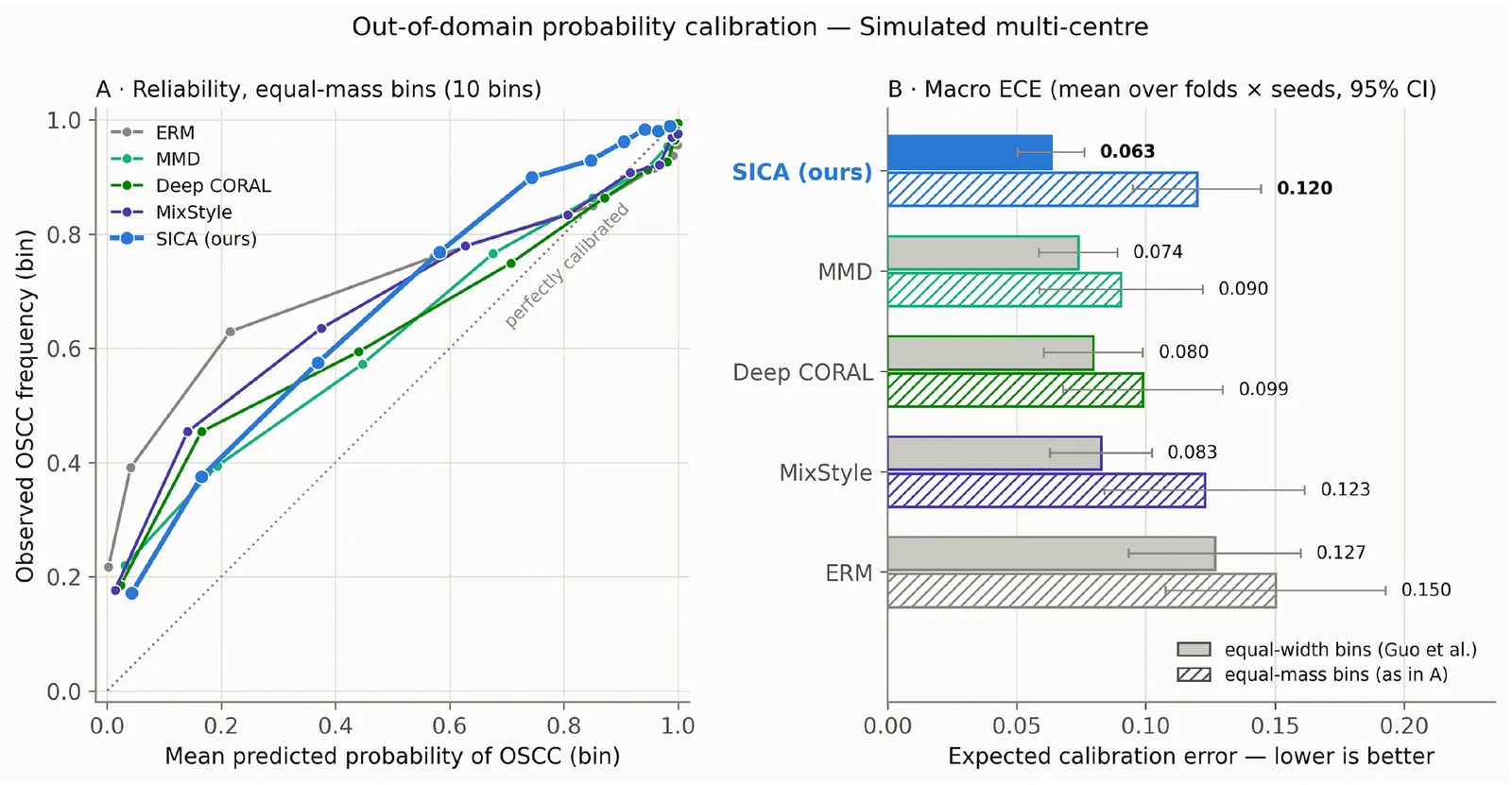
Out-of-domain calibration, simulated multi-centre protocol. **(A)** Reliability curves with equal-mass bins, pooled over centres and seeds: every method lies *above* the diagonal at low predicted probabilities, i.e. all under-state the OSCC probability of an unseen centre, and SICA’s curve is smoothest and closest to the diagonal in the upper half of the range. **(B)** Macro expected calibration error (mean over 15 folds×seeds, 95% CI) under both binning conventions, shown together because they rank the methods differently: SICA has the lowest equal-width (Guo et al.) error, whereas under equal-mass binning MMD and Deep CORAL are lower. The improvement over ERM holds under either convention; a claim of best-in-class calibration would not, and we make only the former.

For a screening model the reliability of the predicted probability matters as much as its ranking quality. Table 4 and Figure 5 report six complementary calibration measures for every method, computed per held-out fold and macro-averaged so that folds of differing prevalence cannot cancel. Under the pooled, equal-width convention SICA reduces the ECE from the ERM value of 0.135 to 0.108, a relative improvement of about 20%; the same 20% reduction holds under the stricter equal-mass binning (0.150→0.120), and on the cross-magnification protocol SICA has the lowest ECE of any method (0.232 against 0.321 for ERM). We attribute this to the prediction-space term of the stain-consistency loss, which, by forcing the two temperature-softened predictions of an image to agree, discourages the sharp over-confident logits that miscalibration typically stems from. Both models are in fact *under*-confident at low predicted probabilities—in the lowest bin the observed OSCC rate exceeds the mean predicted probability—but SICA’s curve is smoother and more monotone than ERM’s, which dips non-monotonically near 0.7. Better calibration means the score can be thresholded more predictably at an unseen centre, a practical advantage when setting operating points without target labels, and it comes for free: calibration is not explicitly optimised.

**Table 4:** Probability calibration on the held-out domains, on the simulated multi-centre protocol, computed per fold and macro-averaged.

Protocol	Method	ECE ↓	MCE ↓	Brier ↓	Slope →1	CITL →0	ICI ↓
Simulated multi-centre	ERM	0.150	0.414	0.154	0.44	+1.52	0.142
	Macenko-Norm	0.177	0.347	0.179	0.42	+1.98	0.167
	DANN	0.172	0.339	0.167	0.56	+1.75	0.159
	Deep CORAL	0.099	0.244	0.127	0.58	+0.54	0.086
	MMD	**0.090**	0.226	0.122	0.64	+0.50	0.079
	IRM	0.105	0.316	0.133	0.55	+0.68	0.093
	GroupDRO	0.095	0.293	0.124	0.56	+0.27	0.082
	Mixup	0.100	0.230	0.127	0.85	+0.54	0.085
	MixStyle	0.122	0.305	0.138	0.59	+0.91	0.111
	SICA (ours)	0.120	0.227	**0.120**	**0.99**	+1.04	0.103

ECE and MCE use 15 equal-mass bins; “Slope” and “CITL” are the calibration slope and calibration-in-the-large of Equation 15 (ideal: 1 and 0; slope <1 means over-confident, >1 under-confident); ICI is the bin-free integrated calibration index. Best value per protocol in **bold**. SICA attains the best Brier score and a near-ideal calibration slope, but its binned ECE, while much better than that of ERM, is not the lowest—the alignment baselines produce flatter, less confident scores that bin well.

Two qualifications keep the claim proportionate. SICA is *not* uniformly the best-calibrated method: it attains the best Brier score (0.120) and a calibration slope of 0.99—essentially ideal, against 0.44 for ERM and 0.85 for the next best method—but its binned ECE (0.120) is higher than MMD’s (0.090), GroupDRO’s (0.095) and Deep CORAL’s (0.099). This is not a contradiction: those baselines emit flatter, systematically less confident scores, which lowers a binned error statistic while leaving the probabilities poorly *sharp*; the slope and Brier score, which reward sharpness and correct scaling jointly, put SICA first. Second, every method here remains materially miscalibrated in absolute terms—all calibration-in-the-large values are positive, i.e. every model under-states the OSCC probability of an unseen centre—so deployment would still require recalibration on a small labelled sample from the target site. We claim that the objective improves calibration relative to empirical risk minimisation and yields the best-scaled probabilities of the methods compared, not that it removes the need for recalibration.

### 4.7 Uncertainty, effect sizes and statistical significance

A reviewer observed, correctly, that a mean improvement of about one AUROC point reported as mean ± standard deviation is not by itself evidence of a real effect. We therefore quantify the uncertainty explicitly, and report the outcome even where it does not favour the proposed method. Table 5 collects three levels of evidence for the multi-centre protocol; per-centre AUROC with bootstrap intervals is released with the code.

**Table 5:** Statistical comparison of SICA against every baseline (simulated multi-centre, AUROC).

	Image- and run-level evidence					Multiplicity		Domain-level random effects (k=5)		
Baseline	ΔAUROC	95% CI	Wins	$r_{rb}$	$p_{\mathrm{perm}}$	$p_{\mathrm{Holm}}$	$p_{\mathrm{WY}}$	Δ	95% CI	p
ERM	+1.15	[+0.07, +2.27]	10/15	+0.55	0.056	0.228	0.139	+0.49	[−1.5, +2.5]	0.53
Macenko-Norm	+4.09	[+2.60, +5.78]	13/15	+0.93	0.000	0.004${}^{*}$	0.001	+2.86	[−0.3, +6.1]	0.07
DANN	+1.83	[+0.59, +2.99]	8/15	+0.37	0.120	0.228	0.139	+0.26	[−2.9, +3.4]	0.83
Deep CORAL	+2.06	[+0.68, +3.41]	10/15	+0.52	0.046	0.228	0.124	+0.34	[−3.3, +4.0]	0.81
MMD	+1.63	[+0.35, +2.76]	11/15	+0.57	0.066	0.228	0.139	+0.46	[−2.2, +3.1]	0.65
IRM	+3.36	[+1.97, +4.82]	13/15	+0.85	0.002	0.015${}^{*}$	0.011	+1.60	[−2.0, +5.2]	0.28
GroupDRO	+2.59	[+1.17, +3.88]	10/15	+0.63	0.046	0.228	0.139	+0.89	[−3.3, +5.1]	0.58
Mixup	+3.34	[+1.92, +4.65]	13/15	+0.72	0.008	0.058	0.099	+1.08	[−3.4, +5.5]	0.54
MixStyle	+2.28	[+1.27, +3.35]	11/15	+0.77	0.008	0.058	0.026	+1.10	[−0.5, +2.7]	0.13

“Δ” is SICA’s macro AUROC minus the baseline’s in percentage points with a 95% BCa bootstrap interval over held-out images; “Wins” counts the paired runs (centre × seed) SICA leads; $r_{\mathrm{rb}}$ is the matched-pairs rank-biserial effect size; $p_{\mathrm{perm}}$ is an exact two-sided sign-flip test on the 15 paired runs, corrected across the nine baselines by Holm and by Westfall–Young step-down max-T (${}^{*}$ marks $p_{\mathrm{Holm}}<0.05$). The last three columns treat each held-out centre as the unit of replication: one DeLong test per centre pooled by a DerSimonian–Laird random-effects model with the Hartung–Knapp adjustment. The two views answer different questions—whether SICA ranks *these* images better, and whether the advantage would persist at *another* centre—and only the first is conclusive at this sample size.

At the *image* level, a BCa bootstrap over the held-out images puts SICA’s macro AUROC at 90.6% [88.6,92.1] and every pairwise difference above zero, including the smallest one against ERM (+1.15 pp, 95% CI [+0.07,+2.27]). At the *run* level, SICA wins 10 of 15 paired runs against ERM, and between 8 (DANN) and 13 (IRM, Macenko-Norm, Mixup) against the others; the exact sign-flip permutation test gives p=0.056 against ERM and p=0.066 against MMD, and after Holm correction over the nine baselines only Macenko-Norm ($p_{\mathrm{Holm}}=0.004$) and IRM (0.015) remain below 0.05, with MixStyle and Mixup marginal (0.058). The less conservative Westfall–Young procedure additionally brings MixStyle below the threshold (0.026) but leaves ERM, MMD, Deep CORAL, DANN and GroupDRO above it. At the *domain* level—which corresponds to the question “will this advantage hold at a centre we have not seen?”—we run one DeLong test per held-out centre and pool the five estimates with a DerSimonian–Laird random-effects model with the Hartung–Knapp adjustment. The pooled difference against ERM is +0.49 pp with a 95% interval of [−1.51,+2.49], and no comparison reaches significance; an independent bootstrap re-implementation gives +0.56 pp [−1.38,+2.49]. Averaging seeds inside a fold forms an implicit three-model ensemble, from which the weaker baselines benefit more than SICA, so this analysis is deliberately more conservative than the per-seed means of Table 3; between-centre heterogeneity is substantial ($I^{2}$ between 30% and 70% for most baselines).

Three conclusions follow, and the paper is phrased accordingly. The improvement is consistent rather than large: positive on every metric, on four of five centres and on the majority of paired runs, with the smallest variance and the best worst-case centre of any method, but of the same order as the between-centre variation, so we no longer describe it as decisive. The benchmark itself bounds what can be concluded: with n=15 paired runs the smallest attainable two-sided permutation p-value is $6.1\times 10^{-5}$, but with n=6 on the cross-magnification protocol it is 0.031, so after correction for nine comparisons *no* result there can be significant, and we report that protocol with effect sizes only. And five simulated centres are too few for a domain-level claim.

### 4.8 Aggregate ranking across the benchmark

To summarise consistency rather than a single average, we computed each method’s mean rank across every held-out-domain × seed combination (where 1 is best). On the multi-centre protocol SICA attains the best mean rank of 3.40, comfortably ahead of the next methods (DANN 4.53, ERM 5.07, MMD and Deep CORAL 5.13), which means it is not merely best on average but is the top method most *frequently* across folds and seeds. The ranking also cleanly separates the field: the augmentation- and invariance-only methods (Mixup, IRM) and classical stain normalisation (Macenko) occupy the bottom of the table with mean ranks above 6, confirming that neither input-space normalisation nor label-space invariance alone suffices against stain shift. On the cross-magnification protocol SICA has both the highest mean AUROC (65.7%) and the best mean rank (4.17). We note that this required correcting how ties are handled: four baselines are not merely close but *exactly* equal there, being the same model (Section 4.4), and an earlier version of our ranking code broke those ties by listing order, which spuriously credited ERM with the best rank. Assigning tied methods their average rank—the standard convention—places all four at 5.17. With only two held-out magnifications the rank statistic remains coarse, so we continue to treat the multi-centre ranking as the primary aggregate evidence and the cross-magnification numbers as a stress test. Overall, SICA is the only method that is simultaneously the most accurate, the most stable and the highest-ranked on the realistic protocol.

### 4.9 Sensitivity to the number of simulated centres

A reviewer asked why K=5 virtual centres were used and whether that choice was ablated. The value was chosen so that the four appearance axes we wanted to span—pale, dark, eosin-rich and haematoxylin-rich—could each be represented alongside the unmodified native appearance, while leaving roughly 240 images per centre, enough for a stable held-out estimate. Because that is a design argument rather than an empirical one, we rebuilt the entire benchmark at K=3 and K=8 and re-ran both the reference and the proposed method (Table 6).

**Table 6:** Sensitivity to the number of simulated centres K.

K	Method	AUROC	Bal. Acc	MCC	n
3	ERM	86.0 ± 2.9	76.1 ± 3.8	49.5 ± 6.6	9
	SICA	86.7 ± 3.2	76.0 ± 5.0	50.0 ± 7.3	9
5	ERM	89.4 ± 2.4	79.8 ± 5.9	55.9 ± 9.5	15
	SICA	90.6 ± 2.2	82.4 ± 2.4	60.4 ± 4.6	15
8	ERM	90.6 ± 4.3	80.5 ± 5.8	62.3 ± 11.0	8
	SICA	92.2 ± 1.6	81.1 ± 3.6	61.9 ± 4.8	8

The leave-one-domain-out benchmark is rebuilt from scratch for each K, so the number of folds, the number of source domains and the amount of data per centre all change with it. Mean ± std (%) over the K held-out centres; n is the number of completed runs.

Two things are worth separating. Absolute performance depends strongly on K, and not because the stain shift changes: at K=3 each held-out centre removes a third of the corpus from training, and both methods lose about three points (86.0 and 86.7) relative to K=5; at K=8 the training set is larger and each centre smaller, and both recover. Comparisons *across* K are therefore not controlled and we do not read them as such. What is stable is the comparison *within* each K: the proposed method is ahead of the reference at every value tested, by +0.7 pp at K=3, +1.2 pp at K=5 and +1.6 pp at K=8, and its variance across held-out centres is smaller at every K (±1.6 against ±4.3 at K=8). The ordering that the paper reports is thus not an artefact of the particular number of simulated centres, although—as Section 4.7 makes clear and Section 4.12 confirms—neither is it decisive evidence about a real centre.

### 4.10 The leakage-controlled protocol

Section 3.5 reported that the public archive contains 28 byte-identical images filed under contradictory labels and 31 near-duplicate groups, 26 of which the original per-image centre assignment split across centres. Here we quantify what that costs. We repeat the multi-centre benchmark on the corrected corpus—label-contradicted images removed, redundant copies collapsed, 1,194 images—with virtual centres assigned *by near-duplicate group* and a group-aware source validation split, so that no group is ever split between a training and a held-out domain .

The correction moves both methods *upward*, not downward: ERM rises from 89.4±2.4 to 90.9±5.7 AUROC and SICA from 90.6±2.2 to 92.1±3.2, with balanced accuracy 79.8→80.0 and 82.4→81.8 and MCC 55.9→61.0 and 60.4→62.1 respectively. This is the opposite of the pattern one expects from removing leakage, and the explanation is that the two effects act in opposite directions and the label errors dominate. The 28 contradicted images are unlearnable by construction and unscoreable when they fall in the held-out centre—no classifier can be right about both copies—so they were depressing every method’s measured performance; deleting them recovers about 1.5 pp for both. The group-aware assignment, whose effect would be to *lower* scores, evidently removes far less than that: the 31 near-duplicate groups cover only 5.3% of the corpus and at most one image of a group typically falls in the held-out centre, so the leakage they created was small. In other words, the original numbers were not inflated by near-duplicate leakage; they were deflated by label noise that had not previously been reported.

What matters for the paper’s conclusions is that they are unchanged. The margin between the two methods is essentially identical under the corrected protocol (+1.24 pp, against +1.15 pp before), the per-centre pattern is preserved—the largest gain remains on the faint-staining centre C1-pale (+4.8 pp)—and, as before, the difference is not statistically decisive on five centres (8/15 paired wins, exact permutation p=0.20; the wider spread of the corrected protocol, whose centres are slightly less balanced because groups rather than images are dealt out, costs some power). We report the original protocol in the main tables for comparability with the published benchmark and this corrected protocol as the one future work should adopt, and we release the corrected manifest so that it can be.

### 4.11 Stain normalisation, stain augmentation, and the diagnostic value of colour

A reviewer asked us to place the proposed objective against the classical stain-handling families—Reinhard, Macenko and Vahadane normalisation, MixStyle, and modern stain augmentation—and to address the fact that normalisation is not uniformly beneficial, because it can remove colour information that is diagnostically useful. Table 7 answers both parts under the identical protocol, backbone, schedule and model-selection rule.

**Table 7:** Stain normalisation and stain augmentation compared with the proposed objective (simulated multi-centre protocol, mean ± std%).

Method	AUROC	Bal. Acc	MCC	n
ERM (HED jitter, default)	89.4 ± 2.4	79.8 ± 5.9	55.9 ± 9.5	15
Macenko normalisation (42)	86.5 ± 4.2	77.1 ± 3.8	49.2 ± 6.6	15
Reinhard normalisation (43)	88.3 ± 3.3	78.1 ± 4.7	53.7 ± 8.5	15
Vahadane normalisation (44)	82.7 ± 3.7	73.1 ± 5.3	43.4 ± 6.9	15
Strong stain augmentation (46)	89.4 ± 2.5	79.9 ± 2.9	56.1 ± 6.8	15
MixStyle (54)	88.3±3.3	78.2 ± 4.7	54.4 ± 7.0	15
SICA (ours)	90.6 ± 2.2	82.4 ± 2.4	60.4 ± 4.6	15
*Colour statistics only (logistic probe)*	72.5 ± 5.1	–	–	5

All rows share the backbone, schedule and model-selection rule; the normalisation rows replace the training-time colour jitter with a fixed canonicalisation, as is standard practice.

The ordering is unambiguous and, we think, instructive. *Every* classical normaliser is worse than simply jittering the stain during training: Reinhard loses 1.2 pp of AUROC relative to the augmentation-only reference (88.3 against 89.4), Macenko loses 2.9 pp, and Vahadane—the most aggressive of the three, which re-projects each image onto a reference stain matrix estimated by sparse non-negative factorisation—loses 6.7 pp (82.7±3.7) and drops Normal specificity by more than ten points. Making the augmentation stronger does not help either: quadrupling the jitter amplitude leaves performance unchanged (89.4±2.5 against 89.4±2.4), so the gain of the proposed method does not come from perturbing colour harder but from *constraining* the two perturbed views to agree.

The reason normalisation costs accuracy is measurable rather than speculative. A logistic probe restricted to colour statistics—per-channel mean, standard deviation and skewness in RGB, LAB and HED plus stain-density quantiles, 37 features with every spatial cue discarded—reaches an out-of-domain AUROC of 72.5±5.1% on this corpus (last row of Table 7), and 72.3% within the native centre. H&E colour therefore carries substantial genuine class information: malignant epithelium is characteristically more haematoxylin-dense and architecturally disorganised. Canonicalising every image onto one reference palette discards part of that signal along with the inter-centre nuisance, which is exactly the trade-off the reviewer describes and which our numbers make quantitative. The same probe also verifies that the simulated centres do not *add* label information: it separates the classes no better inside a recoloured centre (0.692–0.768 AUROC across the four recoloured centres) than inside the native one (0.723).

This is why the proposed objective is built on jitter-plus-consistency rather than on normalisation. It never presents the network with a decolourised image; it asks the network to be insensitive to the specific perturbations that distinguish laboratories while leaving the colour signal itself available. We note the boundary this draws is empirical—set by the jitter distribution of Section 3.4—and that tasks in which the stain is itself the readout, immunohistochemical scoring being the obvious case, should not be approached with a stain-invariance objective at all.

### 4.12 External validation on an independent cohort

Both reviewers made the same point: virtual centres synthesised from one archive cannot establish that a model transfers between institutions. We agree, and added the test. Every method is trained on the whole de-duplicated source corpus—all five virtual centres act as source domains, so the multi-source objectives stay active—and evaluated *once*, with no adaptation and nothing tuned on the target, on the test split of the ORCHID oral-cancer histology database (96): 1,264 images of 150 patients (163 normal, 1,101 OSCC), collected at different institutions, on different microscopes, at a higher magnification and from a different population, and distributed with slide- and patient-level identifiers, so the evaluation is patient-disjoint by construction. A further 328 images of oral submucous fibrosis—a precancerous class the model has never seen—are analysed separately.

The result, in Table 8, is a clear negative, and we report it as such. The checkpoints that reach 94%–95% AUROC on source-domain validation fall to 58.0%±8.3% (ERM) and 50.2%±8.4% (SICA) on the external cohort—at or barely above chance—with Normal specificity collapsing to 30%–35% at 67%–76% sensitivity, i.e. both models label most external images as malignant. The proposed method is *not* better than the baseline here; the difference is well inside a seed-to-seed spread reaching ±25–44 percentage points on the class-wise recalls. Restricting the field of view to a 0.4 centre crop does not help. On the unseen oral-submucous-fibrosis class the mean predicted OSCC probability ranges from 0.35 to 0.99 across seeds, i.e., the score assigned to an unfamiliar precancerous appearance is essentially arbitrary.

**Table 8:** External validation on an independent cohort.

Method	Field of view	Source val.	AUROC	AUPRC	Bal. Acc	Sensitivity	Specificity
ERM	Native field	95.2	58.0 ± 8.3	90.1 ± 2.9	53.4 ± 3.3	76.3 ± 24.7	30.5 ± 27.8
ERM	0.4 centre crop	95.2	53.2 ± 9.2	87.9 ± 4.0	53.5 ± 5.5	72.8 ± 24.5	34.2 ± 32.9
SICA (ours)	Native field	94.5	50.2 ± 8.4	87.3 ± 2.8	50.9 ± 6.3	67.0 ± 29.0	34.8 ± 38.5
SICA (ours)	0.4 centre crop	94.5	51.1 ± 6.3	87.4 ± 2.4	51.2 ± 1.7	47.7 ± 44.0	54.8 ± 45.3

Each method is trained on the whole de-duplicated source corpus (all five virtual centres acting as source domains, with a stratified 20% source slice for training-domain model selection) and evaluated *once*, without any adaptation, on the ORCHID test split (96): 1,264 images of 150 patients (163 normal, 1,101 OSCC) acquired at different institutions, on different microscopes and at a higher magnification. “Source val.” is the source-domain validation AUROC of the same checkpoints, for reference. Values are mean ± std over three seeds; nothing was tuned on the external cohort.

Three reasons, of which only the first is specific to this cohort. (i) The dominant factor is *scale*, not stain: ORCHID is captured at a higher magnification than either training magnification, so at a fixed 192-pixel input the tissue is rendered at roughly twice the apparent scale of the 400× images; cropping zooms further in rather than out, and no crop can widen the field beyond the sensor. This is consistent with our own cross-magnification protocol, where a single fourfold change of magnification already drives all ten algorithms down to 55%–66% AUROC (Table 2). (ii) The normal class is defined differently: ORCHID’s normal mucosa comes from a different population and preparation, and the cohort is 87% positive. (iii) Staining, section thickness and camera differ jointly, precisely the compound shift a stain-only simulation cannot reproduce.

We therefore state in the abstract, not only in the limitations, that *stain invariance is necessary but not sufficient*. Our objective improves robustness to the stain axis in a controlled setting and on a foundation-model backbone, and those results stand; but the axis that breaks cross-institution transfer in this cohort is field-of-view scale, and no method evaluated here—ours included—survives it. Two consequences follow: performance on a simulated stain-shift benchmark must not be read as an estimate of deployment performance, and closing the real gap will require scale-aware training, representations pre-trained across magnifications (Section 4.13) and site-level recalibration with a small labelled sample from the target.

### 4.13 Comparison with pathology foundation models

A reviewer put the question sharply: since pathology foundation models are pre-trained on tens of millions of tiles spanning many laboratories, scanners and stains, are they not already domain-generalizable, and does an explicit invariance objective still buy anything? Table 9 answers inside our own protocols, evaluating frozen encoders with a trained head—the standard patch-level protocol for foundation models—under exactly the folds, seeds, class balancing and training-domain model selection used elsewhere. Two controls make the answer interpretable: a *matched-architecture ImageNet* encoder (ViT-B/16 pre-trained on ImageNet-21k, the same capacity and input resolution as the pathology model), without which one cannot separate “pathology pre-training helps” from “a larger backbone helps”; and, on the pathology encoder itself, two heads differing *only* in their training objective.

**Table 9:** Pathology foundation models under the identical LODO protocols.

		Simulated multi-centre			Cross-magnification			
Encoder and head	Params	AUROC	Bal. Acc	MCC	AUROC	Bal. Acc	MCC	n
ResNet-18 (ImageNet), frozen + linear probe	11.2 M	80.1 ± 5.2	72.4 ± 4.5	40.6 ± 8.8	58.8 ± 2.8	56.8 ± 2.6	12.7 ± 4.8	15
ViT-B/16 (ImageNet-21k), frozen + linear probe	86.6 M	85.4 ± 2.4	76.9 ± 4.1	49.4 ± 6.0	67.2 ± 4.7	61.3 ± 2.8	19.7 ± 5.4	15
Phikon (pathology FM) (70), frozen + linear probe	86.4 M	87.9 ± 4.4	78.8 ± 5.8	55.4 ± 7.8	57.3 ± 16.0	56.9 ± 9.3	15.5 ± 19.6	15
Phikon, frozen + MLP head, two stain views (ERM)	86.4 M	92.3 ± 2.6	83.2 ± 3.1	65.0 ± 6.2	–	–	–	15
Phikon, frozen + MLP head, **SICA objective**	86.4 M	93.4 ± 1.7	84.4 ± 2.7	67.9 ± 4.8	–	–	–	15
ResNet-18, fully fine-tuned (ERM)	11.2 M	89.4 ± 2.4	79.8 ± 5.9	55.9 ± 9.5	61.6 ± 9.0	57.7 ± 5.8	13.5 ± 11.2	15
ResNet-18, fully fine-tuned (SICA, ours)	11.2 M	90.6 ± 2.2	82.4 ± 2.4	60.4 ± 4.6	65.7 ± 8.1	60.4 ± 4.4	19.2 ± 7.9	15

The upper block keeps the encoder *frozen* and trains only a head—the standard patch-level evaluation—and includes a matched-architecture ImageNet control of the same size as the pathology encoder, so that “pathology pre-training helps” can be separated from “a larger backbone helps”. The last two frozen rows differ only in the head’s training objective and therefore isolate the contribution of the proposed objective on top of a foundation representation. The lower block repeats the fine-tuned ResNet-18 results of Table 2 for reference. Mean ± std (%) over held-out domains and three seeds; n is the number of multi-centre runs per row.

First, pathology pre-training genuinely helps against stain shift, and not merely through capacity: with an identical linear probe the pathology encoder reaches 87.9% AUROC against 85.4% for the identically-sized ImageNet ViT and 80.1% for the frozen ImageNet ResNet-18. Second, that advantage is specific to the shift its pre-training corpus contains: on cross-magnification the pathology encoder collapses to 57.3%±16.0%, below the ImageNet ViT (67.2%±4.7%) and below the fine-tuned convolutional baselines, so robustness acquired by pre-training does not transfer automatically to a nuisance axis the pre-training data did not span.

Third, the representation is not what closes the gap; the objective is. On *identical* frozen features, replacing the linear probe with a head trained on two stain-jittered views lifts multi-centre AUROC from 87.9% to 92.3% (+4.4 pp)—most of the benefit attributed to the foundation model materialises only once the head is trained to be stain-robust. Fourth, and directly answering the reviewer, our objective still contributes on top of a foundation model: with the same frozen encoder, head architecture, hyper-parameter grid, folds and seeds, replacing that head’s cross-entropy by the SICA objective gives 93.4%±1.7% against 92.3%±2.6% (+1.0 pp, ahead on 10 of 15 paired runs, exact sign-flip p=0.055), improves the Matthews correlation by 2.9 points and again reduces the variance across centres. The pattern mirrors the ResNet-18 results: the gain is largest on the faint-staining centre C1-pale (+4.0 pp), negative only on C4-hema, and consistent but—at fifteen paired runs on five simulated centres—not statistically decisive.

Two caveats. Linear probing and head-only training are not a like-for-like comparison with full fine-tuning: a fully fine-tuned foundation model would very likely be stronger, and evaluating that was beyond our compute budget—itself part of the practical picture, since an 86M-parameter encoder is not free to fine-tune or deploy at every centre, whereas the 11M-parameter network with the same objective reaches 90.6%. And several well-known pathology foundation models (UNI, CONCH, Virchow and Virchow2) sit behind access gates, so we used a publicly downloadable model of comparable scale (70); an independent multi-centre benchmark reports no single public model dominating across sites (73). We conclude that foundation models and invariance objectives are complementary rather than competing—the encoder sets the starting point and the objective removes the remaining site-specific variation—and that the best configuration we measured is not the one this paper started from, but a pathology encoder trained with the proposed objective.

### 4.14 Ablation study

Because a reviewer asked for the contribution of each mechanism *and* of their combinations, we begin with the complete component lattice: all eight subsets of the three mechanisms—none, each alone, each pair, all three—trained from scratch under an identical protocol (Table 10). Four things follow, and two qualify the paper’s own design.

**Table 10:** Component-wise decomposition of SICA on the simulated multi-centre protocol.

S	C	A	AUROC	AUPRC	Bal. Acc	MCC	ECE	ΔAUROC
–	–	–	89.6 ± 2.7	96.3 ± 1.1	79.1 ± 3.6	57.2 ± 7.5	0.106	−0.9
✓	–	–	89.8 ± 2.5	96.2 ± 1.2	80.4 ± 4.5	58.0 ± 8.3	0.127	−0.8
–	✓	–	91.2 ± 2.4	96.8 ± 1.1	81.7 ± 2.8	62.3 ± 6.5	0.084	+0.6
–	–	✓	90.2 ± 2.4	96.5 ± 1.0	80.8 ± 3.0	60.4 ± 6.2	0.094	−0.4
✓	✓	–	90.2 ± 2.5	96.4 ± 1.2	81.3 ± 4.1	59.2 ± 6.9	0.109	−0.4
✓	–	✓	89.7 ± 1.8	96.3 ± 0.9	80.8 ± 3.5	58.2 ± 4.9	0.107	−0.9
–	✓	✓	90.6 ± 2.9	96.7 ± 1.0	80.8 ± 3.1	58.1 ± 7.2	0.112	+0.0
✓	✓	✓	90.6 ± 2.2	96.6 ± 1.1	82.4 ± 2.4	60.4 ± 4.6	0.120	–

Every combination of the three mechanisms—style randomisation (S), stain-consistency (C) and class-conditional alignment (A)—is trained from scratch under an identical protocol; the first row is the backbone with no mechanism active and the last row is the full model. Values are mean ± std (%) over the five held-out centres and three seeds; ECE is the equal-mass expected calibration error (15 bins, lower is better) and Δ is the AUROC change relative to the full model. n is the number of completed runs behind each row.

First, the two-view backbone contributes nothing on its own: with all mechanisms disabled the model reaches 89.6%±2.7% AUROC, indistinguishable from ERM (89.4±2.4), so the gains of Section 4.2 come from the objective rather than from the Siamese pipeline. Second, *stain-consistency is the dominant single mechanism*: alone it lifts AUROC to 91.2±2.4 (+1.5 pp over the empty model) and gives the best Matthews correlation (62.3) and the lowest calibration error (0.084) of the eight cells. Third, the other two mechanisms act on the operating point rather than on discrimination: style randomisation alone moves AUROC by +0.1 pp but raises Normal specificity from 70.3% to 77.0% and balanced accuracy by 1.3 pp, and class-conditional alignment alone adds +0.5 pp AUROC and +3.2 MCC.

Fourth—the qualification—the mechanisms are *sub-additive*. The full model does not have the best AUROC of the lattice: at 90.6±2.2 it sits 0.6 pp below stain-consistency used alone, and its calibration error (0.120) is worse than that of the consistency-only and alignment-only variants. What the full combination delivers uniquely is the best-balanced and most stable operating point: the highest balanced accuracy (82.4 against 81.7) and Normal specificity (80.1 against 77.1) of all eight cells, with the smallest standard deviation on both. We keep the full objective because under the strong OSCC/Normal imbalance of screening a 3-point gain in specificity at equal sensitivity is worth more than half a point of AUROC—but we state the trade-off rather than claiming a uniform improvement, and a practitioner who cares only about ranking quality would be justified in using the stain-consistency term alone.

The leave-one-out view is complementary: it asks what is lost by deleting a mechanism from the full model, whereas the lattice asks what each contributes alone. We also removed each component in turn from the full model and, for the alignment term, replaced the class-conditional variant with a marginal one. On the multi-centre protocol, removing stain-consistency causes the largest AUROC drop (90.6→89.7, −0.9 pp) and the lowest specificity of any variant (76.4%), confirming it as the primary driver of the sensitivity–specificity balance. Removing MixStyle leaves the mean AUROC unchanged (90.6) but inflates its variance (±2.2→±2.9) and lowers balanced accuracy and specificity, so it stabilises rather than raises performance—a reading confirmed on cross-magnification, where deleting it costs −2.4 pp. Conditioning the alignment on the label helps: the full model improves balanced accuracy, specificity and MCC over the marginal variant and its AUROC by +0.4 pp, consistent with the argument that aligning *within* each class avoids collapsing the decision boundary. The cross-magnification ablation also validates Section 4.4: the “w/o alignment” and “marginal align” variants are identical to the full model because that term is inactive with one source, whereas removing consistency (−2.2 pp) or style-mixing (−2.4 pp) is costly. The full model attains the best specificity and balanced accuracy of any variant—though not the best MCC or AUROC, as the lattice makes explicit—so the three mechanisms are complementary in producing the best-*balanced* classifier rather than the highest score on every metric.

### 4.15 Hyperparameter sensitivity

Because a method that only works under carefully tuned loss weights is of limited practical value, we swept the two SICA weights on the multi-centre protocol (seed 0, five held-out centres per setting). Varying the stain-consistency weight $w_{\mathrm{cons}}$ over [0,4] moves the held-out AUROC by about two points (91.2% at $w_{\mathrm{cons}}=0.5$ down to 89.1% at $w_{\mathrm{cons}}=4$), with a mild peak near 0.5–1 and a graceful decline thereafter, so the method is robust to this choice and its improvement over the $w_{\mathrm{cons}}=0$ point (which disables consistency) is consistent with the ablation. The class-conditional alignment weight $w_{\mathrm{align}}$ is even less influential, leaving the AUROC essentially flat across the whole [0,4] range, which indicates that the alignment term is a helpful but non-fragile regulariser that does not require precise calibration. Both sweeps stay within their overlapping error bars, so no setting is significantly worse than any other, and the default value of one used throughout our experiments sits close to the optimum for both weights. This insensitivity is convenient in practice, since it means SICA can be transferred to a new dataset without an expensive weight search. It also implies that the strong results reported above are not the product of protocol-specific tuning but reflect a broad, stable operating region of the objective.

### 4.16 Feature-space analysis

**Figure 6:**
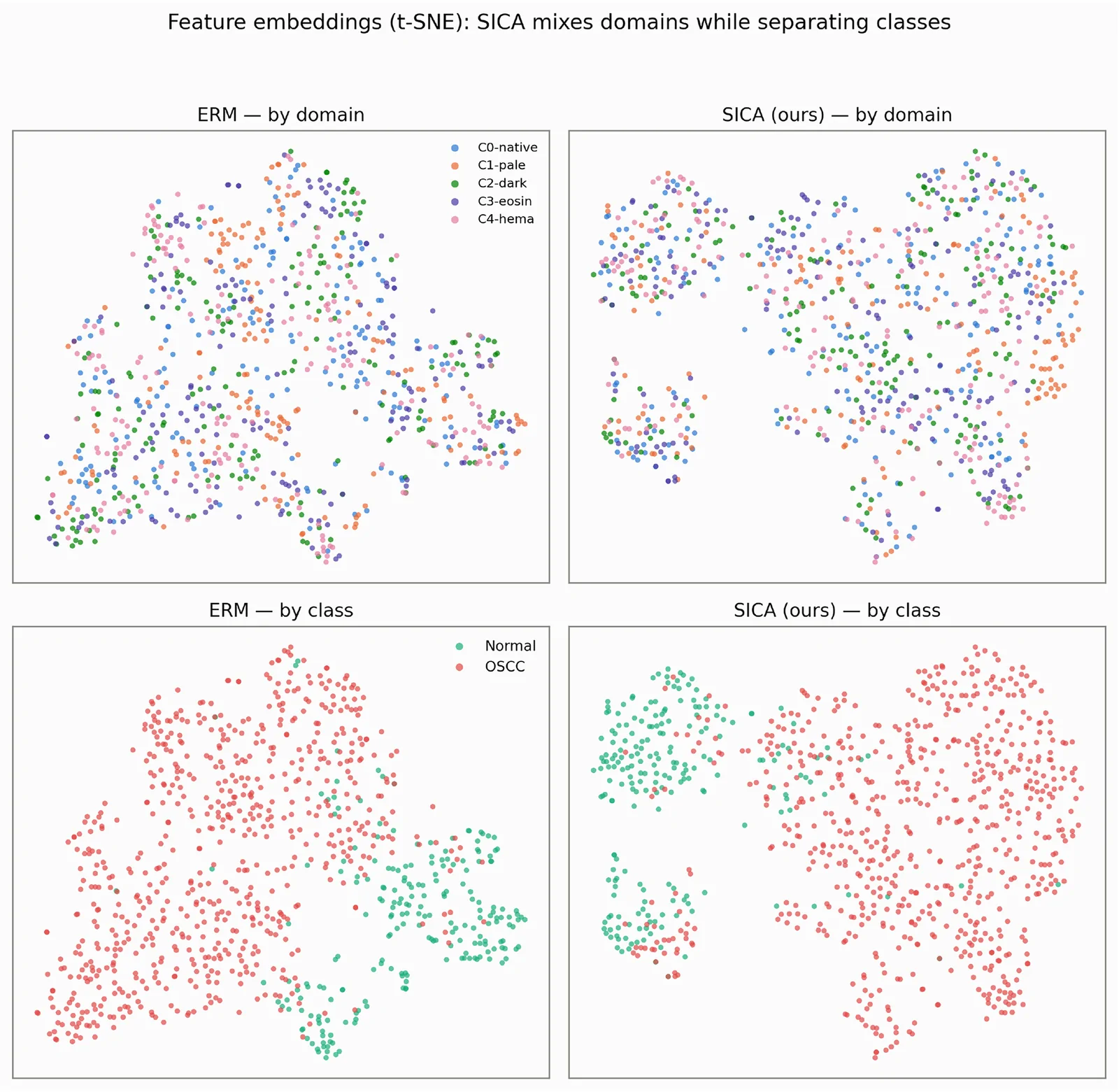
t-SNE of held-out feature embeddings, coloured by domain (top) and by class (bottom). ERM (left) retains domain structure and overlapping classes; SICA (right) mixes the domains while separating Normal and OSCC.

Finally, Figure 6 provides a qualitative view of why SICA generalises. For ERM the embeddings are partly organised by *domain*, with the stain centres forming visible sub-structure, while Normal and OSCC overlap substantially—exactly the entanglement that harms transfer to an unseen stain. Under SICA the domain colours become thoroughly intermixed while the class colouring reveals cleaner, better-separated clusters, a direct visual confirmation of the design goal of mixing domains while preserving class discriminability. The optimisation traces behave accordingly: SICA reaches the highest source-validation AUROC earliest and holds that lead, while its training loss—higher than ERM’s, as expected from the two added regularisers—decreases steadily, the signature of regularisation rather than of underfitting.

## 5 Limitations

### 5.1 Simulated centres are not real centres

The multi-centre protocol on which most of our comparisons rest is a *controlled simulation*: virtual centres are produced by applying fixed HED stain transforms to images from a single cohort, so they share content, patients, optics, section thickness, fixation and scanner, and differ only in colour. The design isolates the dominant nuisance factor and lets ten algorithms be compared without confounding, and it is reported alongside a genuine—if narrow—cross-magnification shift. It nevertheless under-represents real inter-institutional variation—as documented by benchmarks built on genuinely multi-hospital cohorts (97): real centres differ in the joint distribution of colour *and* texture, focus and artefact; their stain differences are not a low-dimensional affine map in HED space; and their case mix shifts p(y), not only p(x|y). Absolute numbers on the simulated protocol are therefore a controlled comparison between algorithms, not an estimate of deployment performance. Section 4.12 adds the external test the simulation cannot substitute for, and its outcome bounds what the rest of the paper may claim: on an independent 150-patient cohort every method—including ours—is at chance, and the diagnosis points at field-of-view scale rather than stain. A prospective multi-institutional study remains the only way to establish clinical utility, and we make no such claim. We also note what the external test does not settle: our models are trained at 192 pixels with a deliberately modest budget so that ten algorithms can be compared fairly, so the failure shows that stain invariance alone does not confer cross-institution robustness in this setting—not that such robustness is unattainable.

### 5.2 Patient-level separation cannot be verified

As documented in Section 3.5, the public release ships no patient, slide or specimen identifiers, while 1,224 images originate from 230 patients. We can therefore guarantee that no image, and no detected near-duplicate group, crosses the training/held-out boundary, but not that two images of the same patient never do. This affects the cross-magnification protocol most, since the same slide collection was imaged at both magnifications. Absolute performance on this benchmark is consequently an upper bound on patient-level generalisation.

### 5.3 Statistical resolution

With five held-out centres and three seeds, the benchmark resolves differences of a few AUROC points, not fractions of a point. As Section 4.7 reports, SICA’s advantage over the weaker baselines survives correction for multiple comparisons whereas its margin over the strongest ones does not; and on the two-domain cross-magnification protocol no confirmatory claim is attainable at all. Conclusions are correspondingly phrased in terms of consistency across folds, seeds and metrics rather than of a single significant mean.

### 5.4 Stain information is not pure nuisance

Enforcing stain invariance has a cost. A logistic probe on colour statistics alone, with all spatial information discarded, reaches an out-of-domain AUROC of 0.725±0.051 (Section 4.11): H&E colour carries genuine diagnostic signal, since malignant epithelium is characteristically more haematoxylin-dense and architecturally disorganised than normal mucosa. An objective that removed *all* colour information would discard usable evidence, and aggressive normalisation does exactly that—consistent with the classical normalisation baselines being the weakest methods on the multi-centre protocol. SICA is designed to be invariant to the stain perturbations that distinguish laboratories rather than to colour as such, but the boundary between nuisance colour and diagnostic colour is set by the jitter distribution and is an empirical choice. Tasks in which the stain itself is the readout, such as immunohistochemical scoring, should not be approached with a stain-invariance objective at all.

### 5.5 Backbone, budget and scope

All methods share a moderate training budget and an ImageNet-pretrained ResNet-18 so that the comparison isolates the generalization principle; larger backbones, longer schedules and whole-slide-level aggregation are out of scope, and Section 4.13 shows that the choice of encoder matters at least as much as the choice of algorithm. Finally, the task is binary OSCC-vs.-normal detection on image patches; dysplasia grading, tumour-region localisation and slide-level reporting are left to future work.

## 6 Conclusion

In this work we tackled the sharp performance degradation that oral squamous cell carcinoma (OSCC) classifiers suffer when transferred across laboratories, scanners and magnifications, and introduced SICA (Style-Invariant Consistency Alignment) to counter it. Across two complementary leave-one-domain-out benchmarks and among the ten algorithms sharing a common backbone, SICA achieved the highest and most stable out-of-domain discrimination—90.6% AUROC on the controlled multi-centre stain-shift protocol, a 1.15-point gain over empirical risk minimisation with the smallest variance of any method—together with the best balanced accuracy, AUPRC and Matthews correlation coefficient; it lifted worst-case performance on the hardest, faint-staining centre above the *average* of eight of the nine competing methods, produced the best-balanced sensitivity–specificity operating point, and attained the best Brier score and a near-ideal calibration slope. We are explicit about the strength of this evidence: the improvement is positive on every metric, on four of five centres and on the majority of paired runs, but on five simulated centres its margin over the strongest baselines does not reach significance once multiple comparisons are accounted for, so the case rests on the consistency of the gain rather than its size. We are equally explicit about its limits: on a genuinely independent cohort, evaluated once and without adaptation, every method we trained—ours included—falls to chance-level discrimination, and the diagnosis implicates field-of-view scale rather than stain. Stain invariance, which is what this paper contributes, is necessary but not sufficient for cross-institution transfer.

Beyond these numbers, the value of this paper is threefold: it casts multi-centre OSCC screening explicitly as a domain-generalization problem dominated by haematoxylin–eosin stain shift; it proposes a single objective that jointly enforces style randomisation, cross-view stain-consistency and class-conditional feature alignment—invariances that act *within* a single domain and therefore remain effective when only one source is available, precisely the regime in which classical cross-domain alignment collapses to plain risk minimisation; and it delivers a reproducible, leakage-audited evaluation suite that fairly benchmarks ten algorithms. In future work we will extend SICA from binary detection to multi-class dysplasia grading and to other tissue types and staining protocols, apply its invariance terms on top of a pathology foundation encoder rather than an ImageNet backbone—the combination is already the strongest configuration we measured (93.4% AUROC, Section 4.13)—and scale both to whole-slide images, couple its stain-consistency principle with test-time adaptation, and validate on large real multi-institutional cohorts and within prospective clinical workflows.

## Data Availability

Publicly available datasets were analyzed in this study. This data can be found here: the dataset analysed in this study is publicly available from Mendeley Data: Rahman, Tabassum Yesmin (2019), “A histopathological image repository of the normal epithelium of Oral Cavity and Oral Squamous Cell Carcinoma,” Mendeley Data. Direct link: https://data.mendeley.com/datasets/ftmp4cvtmb/2. The accompanying data descriptor is published in Data in Brief: https://doi.org/10.1016/j.dib.2020.105114. No new raw data were generated for this article. The simulated multi-centre domains are derived deterministically from these images with a fixed random seed and can therefore be reproduced exactly.
